# Long-term capture and handling effects on body condition, reproduction and survival in a semi-aquatic mammal

**DOI:** 10.1038/s41598-020-74933-w

**Published:** 2020-10-21

**Authors:** Rasmus M. Mortensen, Frank Rosell

**Affiliations:** Department of Natural Sciences and Environmental Health, Faculty of Technology, Natural Sciences, and Maritime Sciences, University of South-Eastern Norway, Bø i Telemark, Norway

**Keywords:** Ecology, Behavioural ecology, Conservation biology, Ecophysiology, Forest ecology

## Abstract

In long-term individual-based field studies, several parameters need to be assessed repeatedly to fully understand the potential fitness effects on individuals. Often studies only evaluate capture stress that appears in the immediate weeks or breeding season and even long-term studies fail to evaluate the long-term effects of their capture procedures. We investigated effects of long-term repeated capture and handling of individuals in a large semi-aquatic rodent using more than 20 years of monitoring data from a beaver population in Norway. To investigate the effects, we corrected for ecological factors and analysed the importance of total capture and handling events, years of monitoring and deployment of telemetry devices on measures related to body condition, reproduction and survival of individual beavers. Body mass of dominant individuals decreased considerably with number of capture events (107 g per capture), but we found no statistically clear short or long-term effects of capture and handling on survival or other body condition indices. Annual litter size decreased with increasing number of captures among older individuals. Number of captures furthermore negatively affected reproduction in the beginning of the monitoring, but the effect decreased over the years, indicating habituation to repeated capture and handling. By assessing potential impacts on several fitness-related parameters at multiple times, we can secure the welfare of wild animal populations when planning and executing future conservation studies as well as ensure ecologically reliable research data.

## Introduction

Studying individuals of a population repeatedly over longer time periods yields substantial scientific insights on age-related changes in various vital rates such as growth^1^, reproductive performance^2^ and survival^3^, but also enables possibilities to connect events and changes at one life-history stage to those of another^4^.

Capture and handling events are expected to induce immediate physiological and behavioural adjustments for the individual to cope with the disturbance, which may result in an intense stress response that can have physical, physiological and behavioural consequences^5,6^. These potential changes in behavioural patterns can affect body condition as well as vital rates and may consequently negatively influence mating success and fitness^7–9^.

To evaluate these potential capture and handling effects, researchers often focus on the immediate harm that could be caused, such as mortalities and physical injuries. Capture and handling methods are often considered efficient as long as the animal survives through the process without noticeable injuries^10^. However, animals often hide symptoms that make them appear vulnerable^11^. Mortality rates and physical injuries at time of capture are not sufficient to successfully assess capture and handling procedures, as the full impact cannot be determined without also evaluating physical, behavioural and physiological effects at multiple times on a long-term scale^12,13^.

When evaluating the more indirect effects of capture and handling in a wide range of species, researchers have, among other things, analysed changes in various body condition indices^9,14–16^, reproductive performance^8,17–22^ and mortality^5,10,23–29^. Most studies only evaluate capture and handling effects using a limited number of parameters associated to, for example, physiology or reproduction, and seldom several categories^30^. However, we might not be able to detect changes in some parameters^22^, or parameters might not be affected despite the level of stress^31^ and animals may habituate to cope with stress^32^. Often effects are only evaluated on a time scale from a couple of weeks to a year after capture, which may relate to limitations of the measured parameters and constraints related to the framework of the project^33^. Even long-term studies that last several seasons are mostly evaluated within each season^30^, although capture and handling may affect individuals at later life stages and across generations^18,34^. To determine the full impact of repeated capture and handling, we need more evidence-based knowledge on physical, behavioural and physiological effects at both short and longer time-scales to understand how capture and handling procedures might affect body condition as well as vital rates that consequently may negatively influence reproductive performance and survival^8,9^.

We use Eurasian beavers (*Castor fiber*) as a model species to study long-term effects of repeated capture and handling in a highly territorial, semi-aquatic mammal. Beavers (including *C. canadensis*) have been intensively studied describing, among other things, reproduction^35–37^, territorial communication^38–41^, resource and habitat use^42–45^ and time-budgets^46–48^. However, effects of capturing and handling individuals, as well as deploying telemetry devices, which have taken place in several of these research projects, have not been investigated besides short-term effects of tagging^16,49^. One would expect these procedures to induce stress responses that may affect short term body condition measures (fat storage in the tail^37^) and behaviour of the beavers. However, these behavioural changes may also affect body condition in the long term and potentially affect the whole population, negatively influencing mating success and survival at several life-history stages and in coming generations^4^.

In our study, we aim to investigate the effects of long-term capture, handling and tagging in a large semi-aquatic rodent, using monitoring data of a beaver population in south-eastern Norway which has experienced repeated capture and handling events since 1997. To investigate the long-term effects, we corrected for ecological factors and analysed the importance of capture and handling events, years of monitoring and deployment of telemetry devices on measures related to body condition, reproduction and survival of individual beavers, all of which ultimately affect fitness of individuals.

## Results

We made on average (mean ± SD) 65.8 ± 8.1 annual captures (median = 63). Kits were captured up to four times (mean = 1.1 ± 1.1, median = 1). Captured yearlings experienced up to five captures (mean = 1.9 ± 1.4, median = 2) and subadults up to seven captures (mean = 3.0 ± 1.7, median = 3), including captures in previous life stages. Adult beavers were captured up to twenty-five times in their lifetime (mean = 5.1 ± 2.3, median = 4). Dominant adults experienced more captures than subordinates (mean_dominant_ = 6.3 ± 2.5, median_dominant_ = 5, mean_subordinate_ = 2.6 ± 1.6, median_subordinate_ = 2) and males and females were captured equally (mean_males_ = 4.4 ± 2.1, median_males_ = 3, mean_females_ = 4.1 ± 2.0, median_females_ = 3).

### Body condition indices

**Figure 1:**
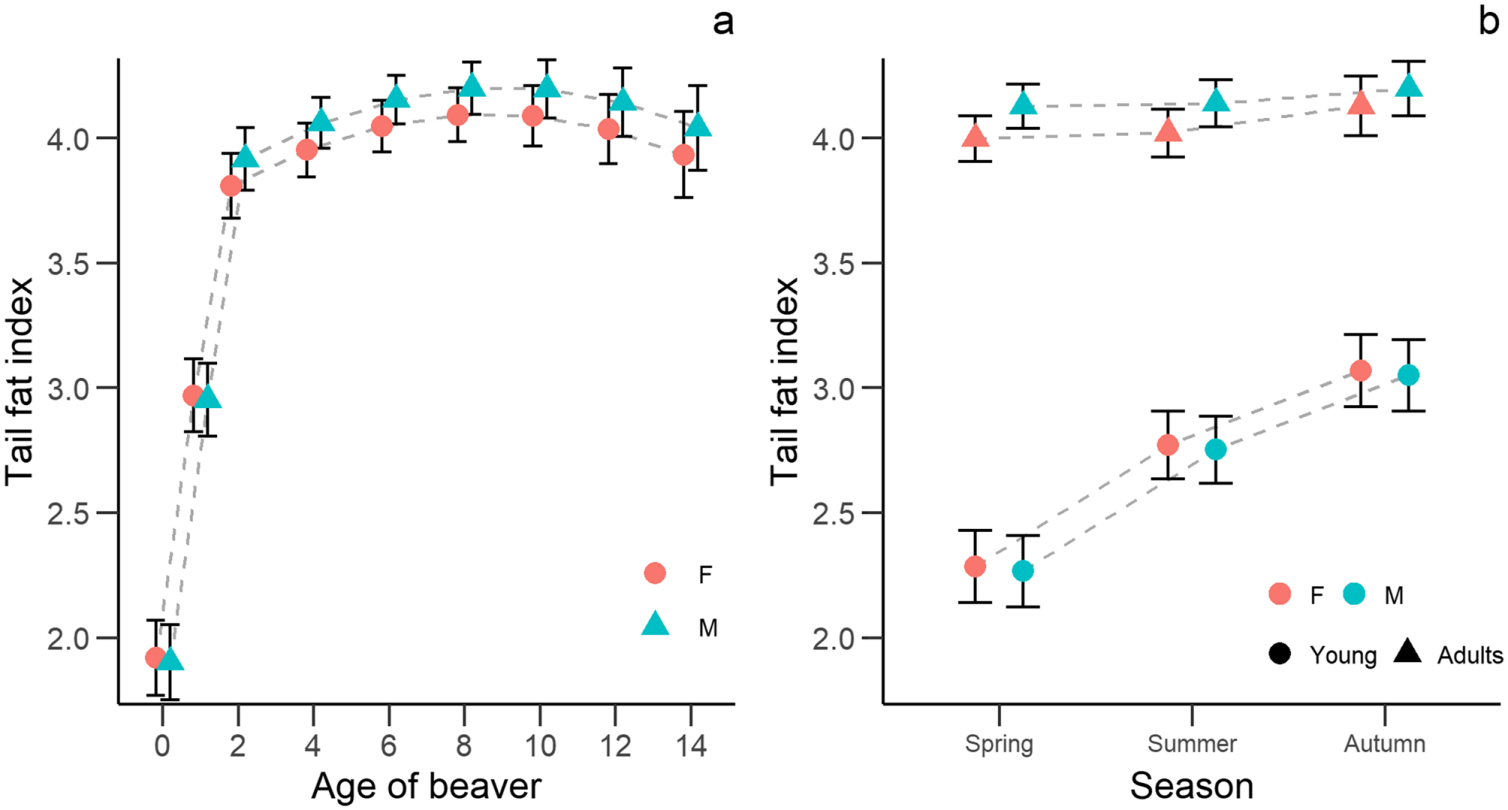
The predicted relationship ± 95% confidence interval between age, sex and tail fat index (**a**) and age, sex, season and tail fat index (**b**) in a Eurasian beaver population in south-eastern Norway between 1998 and 2018.

We found no statistically clear effects of the number of capture and handling events, years of monitoring or from carrying telemetry devices on the variability of the tail fat index in our population. Tail fat index varied clearly with capture season and age among both young and adult individuals (Table 1). It increased clearly over time among individuals, resembling increase in body growth. Among adult individuals, tail fat index increased up to approximately eight years, after which it decreased (Fig. 1). Furthermore, we found statistically clear intra-annual variation in tail fat index among adults with tail fat clearly increasing between spring and autumn (Table 1, Fig. 1). No statistically clear differences were found among young males and females, but adult females had smaller tail fat index than males (Table 1).

**Table 1:** Effect size (β), standard error (SE), lower (LCI) and upper (UCI) 95% confidence interval of explanatory variables for the analysis of tail fat index in a Eurasian beaver population in south-eastern Norway between 1998 and 2018 (n_young_ = 333, n_adults_ = 828).

Variables	Estimate	SE	LCI	UCI	R^2^_marginal_	R^2^_conditional_
**Young (kits and yearlings)**						
Intercept	**1.496**	**0.089**	**1.322**	**1.670**	0.56	0.73
Captures	0.001	0.014	− 0.026	0.028		
Years of monitoring	0.002	0.004	− 0.006	0.010		
Sex (male)	− 0.018	0.037	− 0.090	0.054		
Age	**1.051**	**0.050**	**0.952**	**1.150**		
Season (summer)	**0.486**	**0.061**	**0.366**	**0.606**		
Season (autumn)	**0.784**	**0.068**	**0.651**	**0.917**		
Log (territory size)	− 0.079	0.051	− 0.179	0.021		
Family group size	− 0.003	0.008	− 0.019	0.013		
**Adults (2 + years)**						
Intercept	**3.641**	**0.107**	**3.431**	**3.852**	0.12	0.48
Captures	− 0.005	0.006	− 0.017	0.007		
Years of monitoring	0.000	0.002	− 0.004	0.003		
Carried telemetry device (yes)	− 0.006	0.021	− 0.047	0.034		
Sex (male)	**0.131**	**0.038**	**0.055**	**0.206**		
Age	**0.100**	**0.022**	**0.058**	**0.142**		
Age^2^	− **0.006**	**0.001**	− **0.008**	− **0.004**		
Social status (subordinate)	− 0.145	0.079	− 0.299	0.009		
Origin (resident)	− 0.001	0.016	− 0.033	0.031		
Season (summer)	0.022	0.032	− 0.040	0.084		
Season (autumn)	**0.131**	**0.052**	**0.029**	**0.233**		
Log (territory size)	− 0.008	0.018	− 0.043	0.028		
Family group size	0.001	0.004	− 0.006	0.008		
Captures: social status (subordinate)	0.000	0.003	− 0.006	0.006		
Season (summer): sex (male)	− 0.011	0.035	− 0.080	0.059		
Season (autumn): sex (male)	− 0.061	0.074	− 0.206	0.083		
Social status (subordinate): age	0.018	0.011	− 0.005	0.040		

Beaver ID, capture year and river were included as random effects. We performed model averaging of best models (ΔAICc < 4) to estimate the effect size of each variable. Informative parameters are given in bold.

Reference level of sex: female.

Reference level of season: spring.

Reference level of carried telemetry device: no.

Reference level of social status: dominant.

Reference level of origin: immigrant.

**Figure 2:**
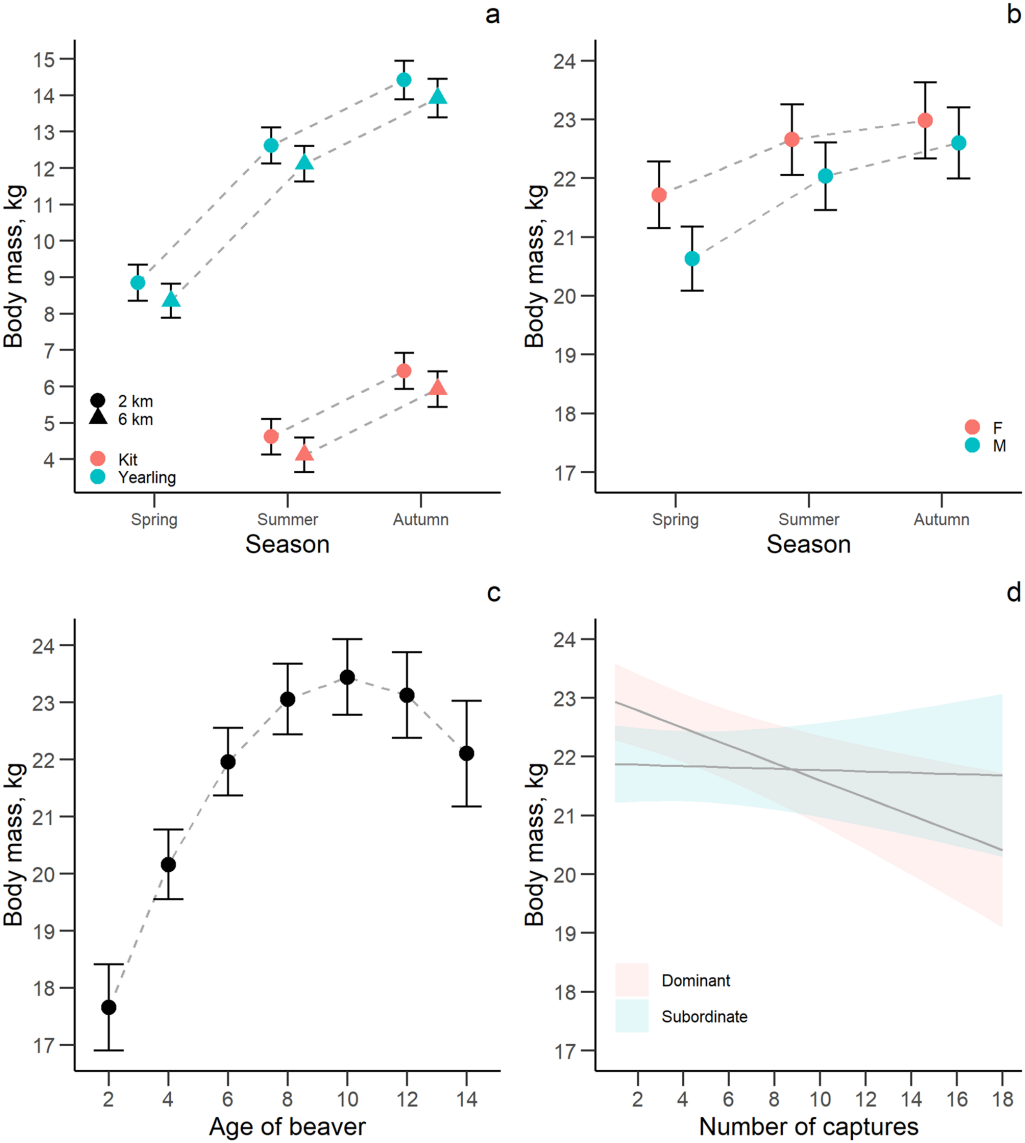
The predicted relationship ± 95% confidence interval between age, territory size and body mass in young beavers (**a**), between season, sex and body mass in adult beavers (**b**), between age, and body mass in adult beavers (**c**) and between number of captures, social status and body mass in adult beavers (**d**) in a Eurasian beaver population in south-eastern Norway between 1998 and 2018.

We found a statistically clear negative effect of capture events on the body mass of dominant individuals, but found no statistically clear effects on other individuals, of years of monitoring, or from carrying telemetry devices (Table 2, Fig. 2). Body mass increased clearly within the year from spring to autumn and with increasing age up to approximately 10 years (Fig. 2). Young individuals from larger territories had smaller body weights than young individuals from smaller territories (Table 2, Fig. 2). As expected, dominant beavers had larger body mass than subordinates (Table 2, Fig. 2). We found no statistically clear differences between young males and females, but adult females were clearly bigger than males in spring and summer (Table 2, Fig. 2).

**Table 2:** Effect size (β), standard error (SE), lower (LCI) and upper (UCI) 95% confidence interval of explanatory variables for the analysis of body mass in a Eurasian beaver population in south-eastern Norway between 1998 and 2018 (n_young_ = 340, n_adults_ = 916).

Variables	Estimate	SE	LCI	UCI	R^2^_marginal_	R^2^_conditional_
**Young (kits and yearlings)**						
Intercept	**4.673**	**0.256**	**4.172**	**5.174**	0.87	0.93
Captures	0.078	0.109	− 0.135	0.291		
Years of monitoring	0.011	0.020	− 0.028	0.050		
Sex (male)	− 0.010	0.076	− 0.160	0.140		
Age	**7.997**	**0.201**	**7.603**	**8.391**		
Season (summer)	**3.765**	**0.204**	**3.364**	**4.166**		
Season (autumn)	**5.570**	**0.236**	**5.108**	**6.033**		
Log (territory size)	− **0.453**	**0.143**	− **0.734**	− **0.172**		
Family group size	− 0.087	0.049	− 0.183	0.010		
**Adults (2 + years)**						
Intercept	**21.983**	**0.283**	**21.428**	**22.538**	0.48	0.78
Captures	− **0.148**	**0.044**	− **0.235**	− **0.062**		
Years of monitoring	− 0.012	0.028	− 0.067	0.043		
Carried telemetry device (yes)	0.003	0.065	− 0.126	0.131		
Sex (male)	− **1.084**	**0.253**	− **1.581**	− **0.587**		
Age	**0.684**	**0.047**	**0.591**	**0.777**		
Age^2^	− **0.088**	**0.007**	− **0.102**	− **0.075**		
Social status (subordinate)	− **0.539**	**0.179**	− **0.889**	− **0.189**		
Origin (resident)	0.001	0.096	− 0.186	0.188		
Season (summer)	**0.939**	**0.205**	**0.537**	**1.341**		
Season (autumn)	**1.265**	**0.237**	**0.801**	**1.730**		
Log (territory size)	− 0.006	0.058	− 0.120	0.108		
Family group size	− 0.059	0.042	− 0.141	0.023		
Captures: years of monitoring	0.010	0.006	− 0.002	0.022		
Captures: social status (subordinate)	**0.137**	**0.042**	**0.055**	**0.219**		
Captures: age	− 0.016	0.009	− 0.033	0.001		
Season (summer): sex (male)	0.462	0.268	− 0.064	0.987		
Season (autumn): sex (male)	**0.703**	**0.317**	**0.082**	**1.324**		
Social status (subordinate): age	0.010	0.035	− 0.059	0.079		

Beaver ID, capture year and river were included as random effects. We performed model averaging of best models (ΔAICc < 4) to estimate the effect size of each variable. Informative parameters are given in bold.

Reference level of sex: female.

Reference level of season: spring.

Reference level of carried telemetry device: no.

Reference level of social status: dominant.

Reference level of origin: immigrant.

**Figure 3:**
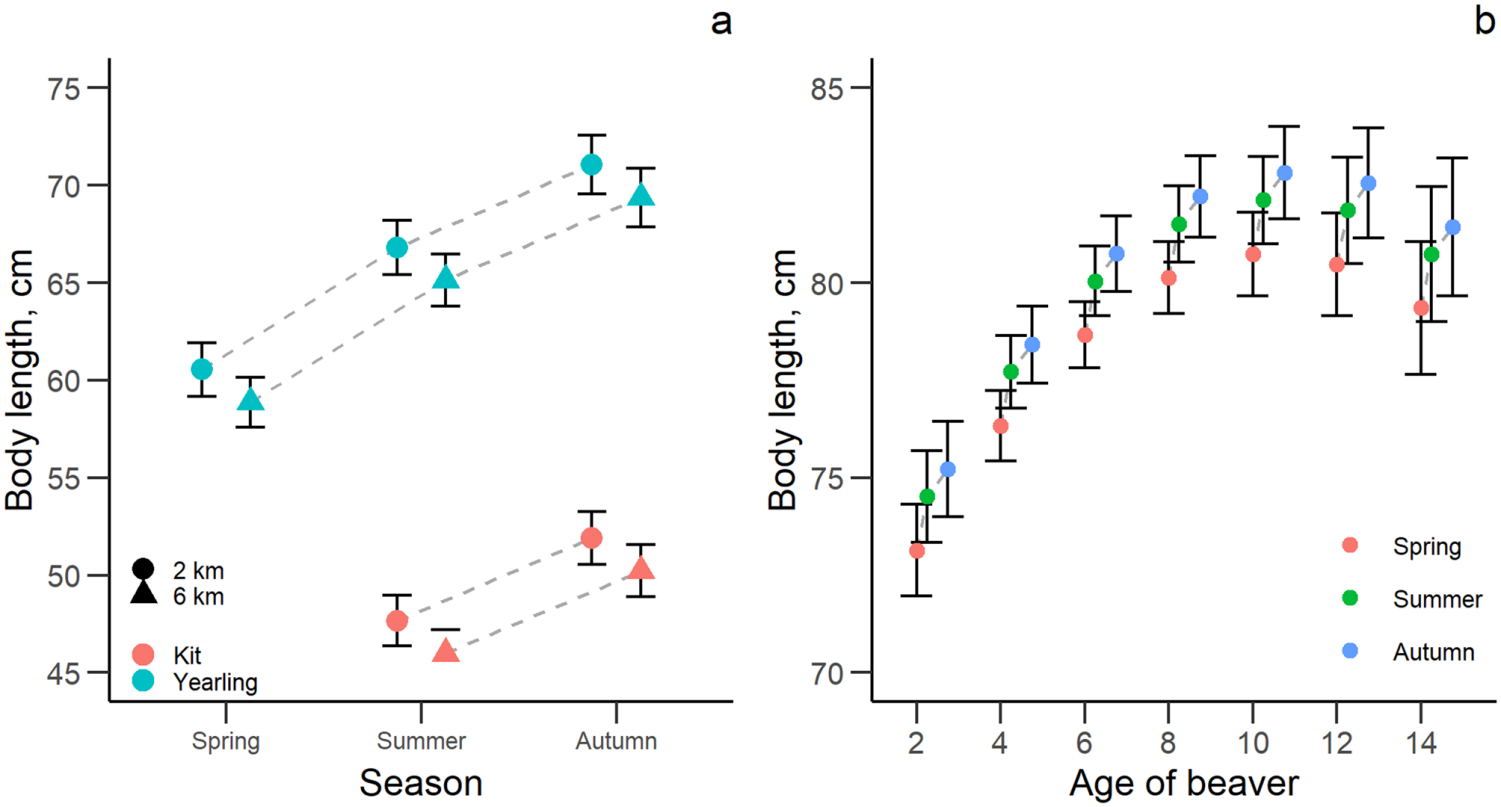
The predicted relationship ± 95% confidence interval between age, territory size and body length in young beavers (**a**) and between age, season and body length in adult beavers (**b**) in a Eurasian beaver population in south-eastern Norway between 1998 and 2018.

No statistically clear effects were found of either number of capture and handling events, years of monitoring or carrying telemetry devices on body length (Table 3). Body length increased clearly within the year from spring to autumn and with increasing age up to approximately 10 years (Fig. 3). Young individuals from larger territories were smaller than young individuals from smaller territories. We found no statistically clear differences in body size among sexes or social rank.

**Table 3:** Effect size (β), standard error (SE), lower (LCI) and upper (UCI) 95% confidence interval of explanatory variables for the analysis of body size in a Eurasian beaver population in south-eastern Norway between 1998 and 2018 (n_young_ = 333, n_adults_ = 829).

Variables	Estimate	SE	LCI	UCI	R^2^_marginal_	R^2^_conditional_
**Young (kits and yearlings)**						
Intercept	**40.410**	**0.880**	**38.686**	**42.134**	0.78	0.84
Captures	0.018	0.199	− 0.372	0.408		
Years of monitoring	0.046	0.065	− 0.082	0.173		
Sex (male)	0.058	0.288	− 0.507	0.622		
Age	**19.164**	**0.612**	**17.965**	**20.364**		
Season (summer)	**6.274**	**0.715**	**4.874**	**7.675**		
Season (autumn)	**10.511**	**0.801**	**8.940**	**12.082**		
Log (territory size)	− **1.528**	**0.475**	− **2.459**	− **0.598**		
Family group size	− 0.103	0.141	− 0.379	0.174		
**Adults (2 + years)**						
Intercept	**78.953**	**0.374**	**78.220**	**79.686**	0.35	0.56
Captures	− 0.018	0.051	− 0.117	0.081		
Years of monitoring	0.023	0.036	− 0.048	0.094		
Carried telemetry device (yes)	− 0.572	0.413	− 1.382	0.238		
Sex (male)	− 0.110	0.246	− 0.592	0.371		
Age	**0.826**	**0.095**	**0.641**	**1.012**		
Age^2^	− **0.108**	**0.011**	− **0.131**	− **0.086**		
Social status (subordinate)	0.072	0.319	− 0.553	0.698		
Origin (resident)	0.105	0.263	− 0.411	0.620		
Season (summer)	**1.379**	**0.257**	**0.876**	**1.883**		
Season (autumn)	**2.077**	**0.319**	**1.451**	**2.703**		
Log (territory size)	− 0.039	0.135	− 0.304	0.225		
Family group size	− 0.081	0.076	− 0.230	0.068		
Captures: social status (subordinate)	0.023	0.063	− 0.100	0.146		
Social status (subordinate): age	0.232	0.128	− 0.019	0.483		

Beaver ID, capture year and river were included as random effects. We performed model averaging of best models (ΔAICc < 4) to estimate the effect size of each variable. Informative parameters are given in bold.

Reference level of sex: female.

Reference level of season: spring.

Reference level of carried telemetry device: no.

Reference level of social status: dominant.

Reference level of origin: immigrant.

### Reproduction

Between 1998 and 2018, we observed 65 dominant females, which produced 1–4 kits in reproducing years. They reproduced on average every 2.5 ± 0.6 years during the duration of their territory occupancy.

**Figure 4:**
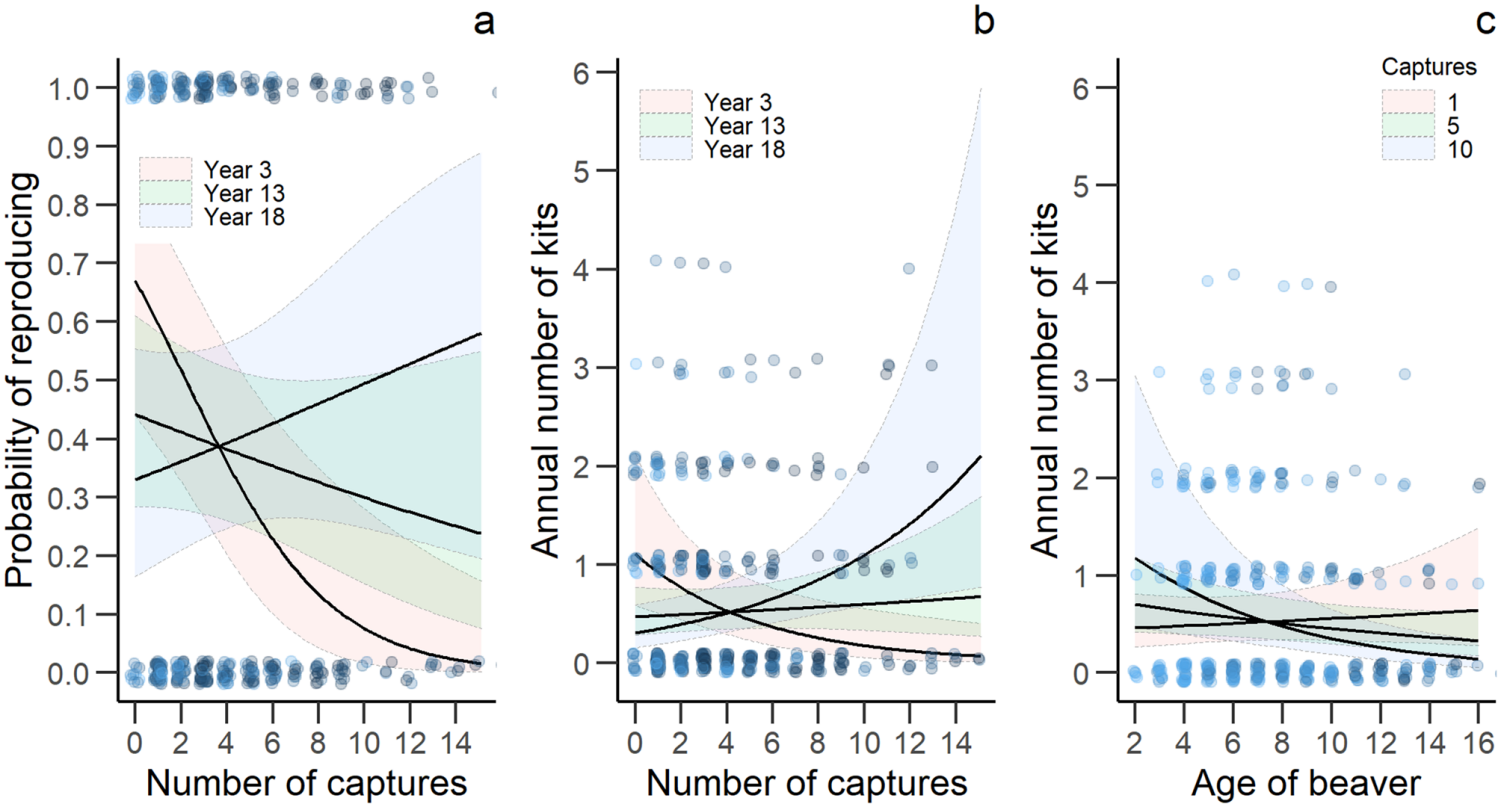
The predicted relationship ± 95% confidence interval between number of captures, years of monitoring and probability of reproducing (**a**) and annual litter size (**b**), and between number of captures, age and probability of reproducing (**c**) in a Eurasian beaver population in south-eastern Norway between 1998 and 2018. Points represent the actual observations with small random variation for better visualisation. Darker colours increase with (**a**,**b**) increasing years of monitoring and (**c**) increasing number of captures.

Number of capture and handling events and years of monitoring had statistically clear effects on both yearly reproduction and annual number of kits produced among females in our population (Table 4, Fig. 4). An increasing number of capture events seemed to have a negative effect in the early monitoring years on both yearly reproduction and annual number of offspring. However, this effect became less clear over the years of the monitoring program. Furthermore, we found a statistically clear negative effect of number of capture and handling events on annual number of offspring produced by older beavers (Table 4, Fig. 4). We found no statistically clear differences among the other investigated variables.

**Table 4:** Effect size (β), standard error (SE), lower (LCI) and upper (UCI) 95% confidence interval of explanatory variables for the analysis of reproduction in a Eurasian beaver population in south-eastern Norway between 1998 and 2018 (n = 388).

Variables	Estimate	SE	LCI	UCI	R^2^_marginal_	R^2^_conditional_
**Probability of reproducing**						
Intercept	− **0.669**	**0.309**	− **1.274**	− **0.063**	0.08	0.14
Captures	− 0.100	0.056	− 0.210	0.010		
Years of monitoring	0.018	0.034	− 0.049	0.085		
Carried telemetry device (yes)	0.232	0.335	− 0.425	0.889		
Log (age)	− 0.072	0.233	− 0.530	0.385		
Origin (resident)	− 0.083	0.227	− 0.528	0.363		
Log (territory size)	0.399	0.263	− 0.117	0.914		
Family group size	0.019	0.047	− 0.074	0.111		
Reproduced previous year (yes)	0.131	0.232	− 0.325	0.586		
Captures: years of monitoring	**0.026**	**0.009**	**0.009**	**0.043**		
Captures: log(age)	− 0.060	0.095	− 0.247	0.127		
**Annual litter size**						
Intercept	− **0.620**	**0.225**	− **1.062**	− **0.179**	0.09	0.21
Captures	− 0.007	0.040	− 0.084	0.071		
Years of monitoring	0.004	0.025	− 0.045	0.053		
Carried telemetry device (yes)	0.049	0.143	− 0.231	0.329		
Log (age)	− 0.041	0.030	− 0.099	0.017		
Origin (resident)	− 0.185	0.250	− 0.674	0.305		
Log (territory size)	0.196	0.179	− 0.155	0.548		
Family group size	0.007	0.023	− 0.039	0.053		
Reproduced previous year (yes)	0.046	0.108	− 0.167	0.259		
Captures: years of monitoring	**0.021**	**0.006**	**0.010**	**0.032**		
Captures: log(age)	− **0.019**	**0.007**	− **0.034**	− **0.005**		

Beaver ID, monitoring year and river were included as random effects. We performed model averaging of best models (ΔAICc < 4) to estimate the effect size of each variable. Informative parameters are given in bold.

Reference level of carried telemetry device: no.

Reference level of origin: immigrant.

Reference level of reproduced previous year: no.

### Survival

**Figure 5:**
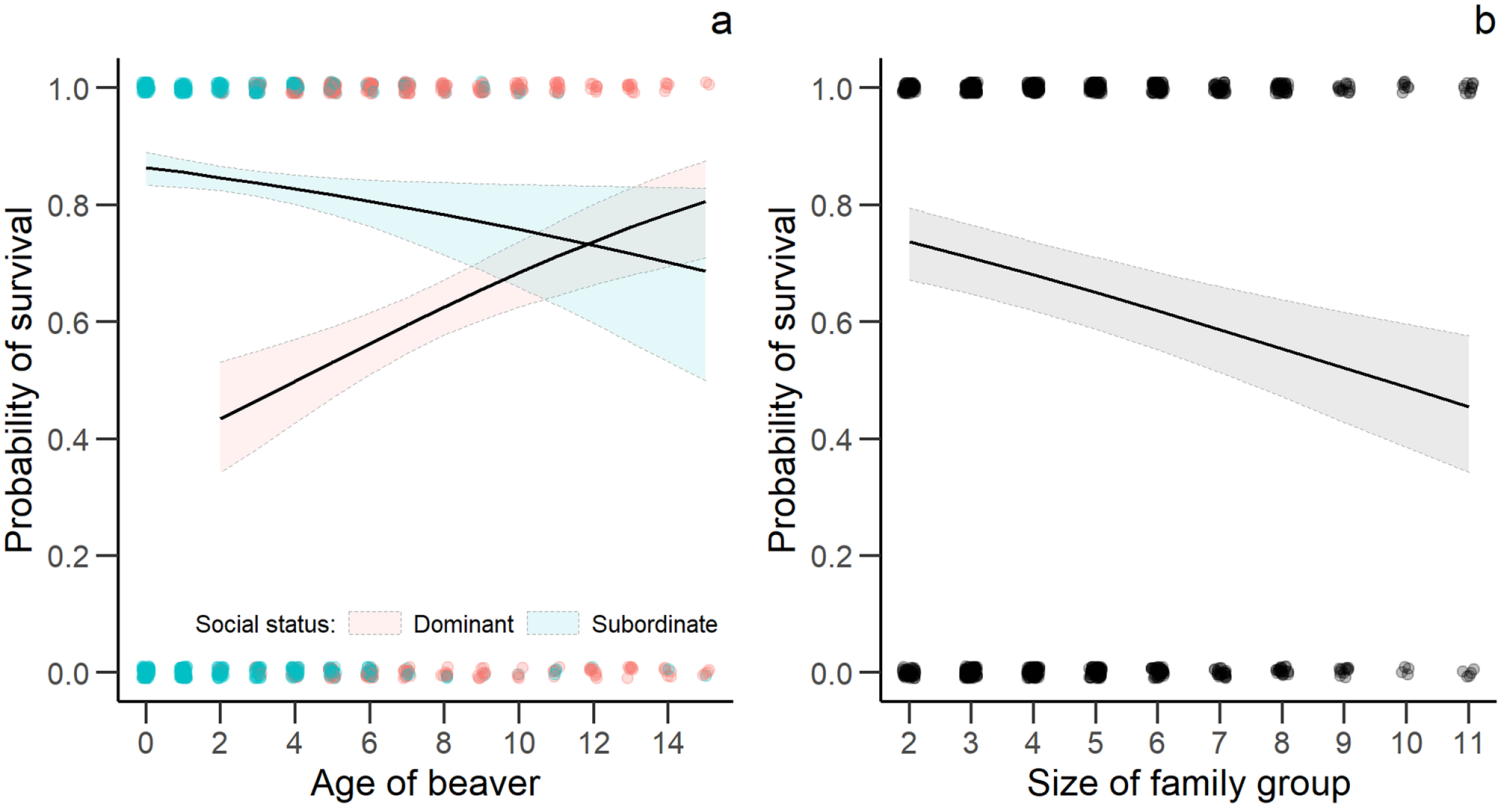
The predicted relationship ± 95% confidence interval between age, social status and survival probability (**a**) and between family group size and survival probability (**b**) in a Eurasian beaver population in south-eastern Norway between 1998 and 2018. Points represent the actual observations with small random variation for better visualisation.

No statistically clear effects were found of number of capture and handling events, years of monitoring or carrying telemetry devices on annual survival (Table 5). Only social status, age and family group size had statistically clear effects on annual survival (Table 5). We found a clear increasing probability of annual survival with age for dominant individuals, whereas annual survival decreased with age for subordinates and with increasing family group size (Fig. 5).

**Table 5:** Effect size (β), standard error (SE), lower (LCI) and upper (UCI) 95% confidence interval of explanatory variables for the continuous time capture-recapture analysis of annual survival in a Eurasian beaver population in south-eastern Norway between 1998 and 2018 (n = 1145).

Variables	Estimate	SE	LCI	UCI	Tjur’s R^2^
**Probability of survival**					
Intercept	− **2.372**	**0.200**	− **2.765**	− **1.980**	0.07
Age	**0.111**	**0.039**	**0.035**	**0.187**	
Social status (subordinate)	**0.857**	**0.230**	**0.407**	**1.307**	
Family group size	− **0.091**	**0.035**	− **0.159**	− **0.022**	
Social status (subordinate): age	− **0.201**	**0.054**	− **0.307**	− **0.094**	

Informative parameters are given in bold.

Reference level of Social status: Dominant.

**Figure 6:**
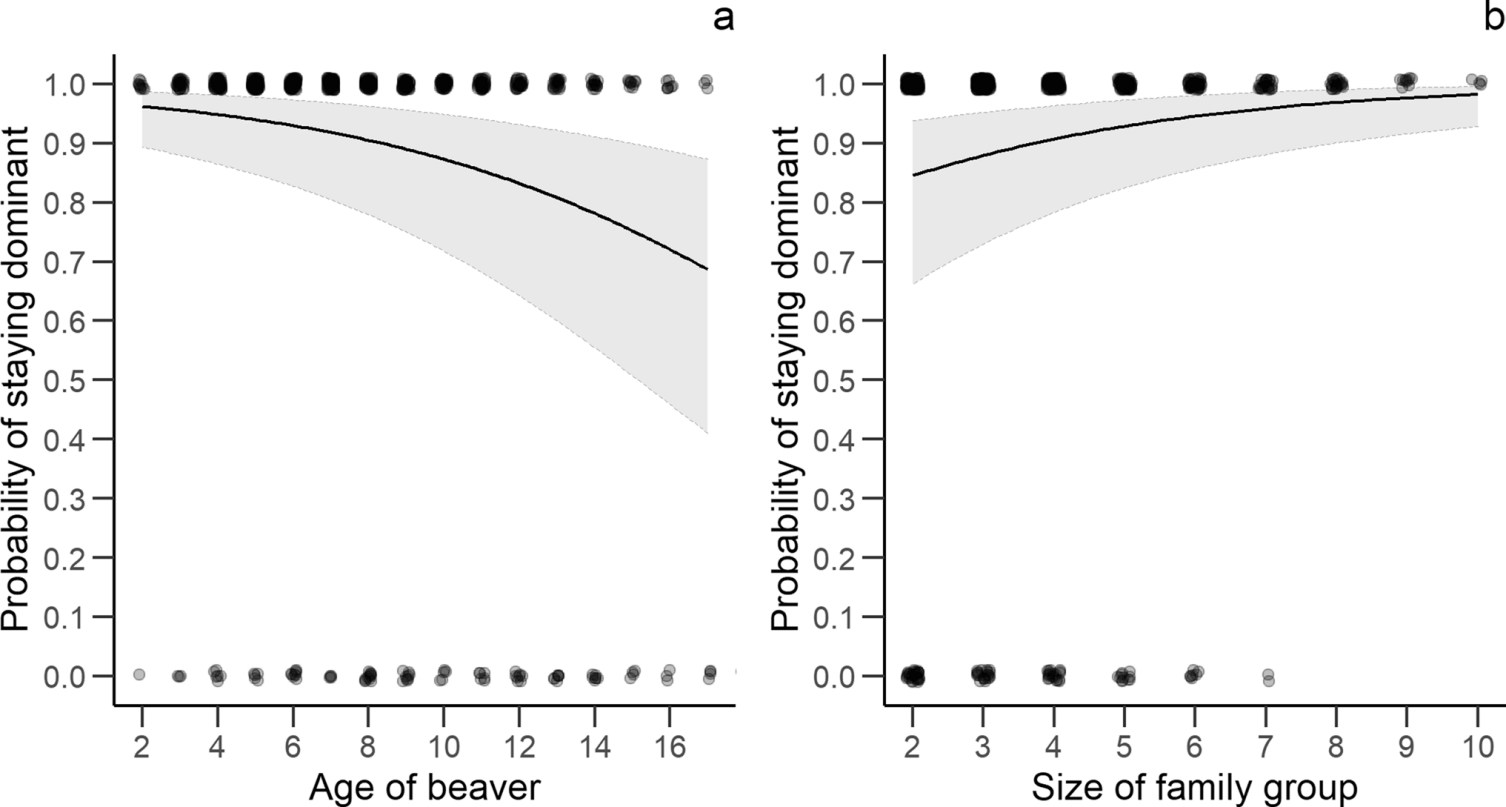
The predicted relationship ± 95% confidence interval between age (**a**), family group size (**b**) and probability of keeping dominance in a Eurasian beaver population in south-eastern Norway between 1998 and 2018. Points represent the actual observations with small random variation for better visualisation.

We found no statistically clear effects of number of capture and handling events, years of monitoring or carrying telemetry devices on the probability of staying dominant in a territory the following year (Table 6). We found a decreasing probability of staying dominant with increasing age and an increasing probability with increasing family group size (Table 6, Fig. 6). No statistically clear differences were found for other investigated variables.

**Table 6:** Effect size (β), standard error (SE), lower (LCI) and upper (UCI) 95% confidence interval of explanatory variables for the analysis of staying dominant the following year in a Eurasian beaver population in south-eastern Norway between 1998 and 2018 (n = 773).

Variables	Estimate	SE	LCI	UCI	R^2^_marginal_	R^2^_conditional_
**Probability of staying dominant the following year**						
Intercept	**3.637**	**0.428**	**2.797**	**4.476**	0.19	0.25
Captures	0.020	0.059	− 0.095	0.136		
Years of monitoring	− 0.062	0.040	− 0.140	0.016		
Carried telemetry device (yes)	− 0.054	0.195	− 0.436	0.328		
Sex (male)	0.017	0.101	− 0.181	0.215		
Age	− **0.164**	**0.037**	− **0.236**	− **0.091**		
Origin (resident)	− 0.101	0.216	− 0.523	0.322		
log(territory size)	− 0.441	0.251	− 0.932	0.050		
Family group size	**0.291**	**0.085**	**0.124**	**0.458**		
Captures: years of monitoring	0.001	0.004	− 0.007	0.008		
Captures: carried telemetry device (yes)	0.004	0.031	− 0.058	0.065		
Captures: age	− 0.002	0.005	− 0.012	0.009		

Beaver ID, monitoring year and river were included as random effects. We performed model averaging of best models (ΔAICc < 4) to estimate the effect size of each variable. Informative parameters are given in bold.

Reference level of sex: Female.

Reference level of carried telemetry device: No.

Reference level of origin: Immigrant.

## Discussion

By evaluating at multiple points in time on a long-term scale, how more than twenty years of repeated capture and handling of individuals in a beaver population affect body condition, reproductive performance and survival, we thoroughly investigate capture and handling effects on important vital rates that ultimately influence fitness of the animals^4^. We found statistically clear effects of repeated capture and handling on body mass of dominant individuals, but found no statistically clear effects of repeated capture and handling on any of the other body condition indices or annual survival probability. However, we observed statistically clear changes through the years in reproductive performance of dominant females, clarifying the importance of investigating several parameter categories when evaluating the consequences of capturing and handling individual animals^9,12,13^. To fully understand fitness effects of capture procedures, one should repeatedly evaluate capture and handling effects at several points in times. Often studies only evaluate capture stress that appears in the immediate days, weeks or the following breeding season after handling^30^. Even long-term studies that investigate capture and handling effects for several years sometimes fail to evaluate long-term effects of their procedures, since they only evaluate short-term effects within each year and season^50–52^. But as we show, capture effects might also have longer-term consequences for individuals, indicating the need for repeated evaluations of condition and fitness. However, this may prove unviable because of limited timeframes or other constraints within the framework of the project that may challenge such long-term continuity^33^.

### Body condition

Body condition indices resemble snapshots of the physiological state of an animal, indicating e.g. past foraging success or ability to cope with environmental pressures^14,53^, which may impact other life-history stages, future reproductive success, and ultimately evolutionary fitness^54,55^. We therefore expected to find variations in size and mass in our population related to number of capture and handling events experienced by each individual, as well as a development over the years of the Norwegian Beaver Project (NBP) as an indication of experienced long-term stress. While several studies have observed negative effects of repeated captures on body condition, body mass and length, only few studies have looked at long-term effects^30^. In long-term studies, up to 21 years, bears that experienced more recaptures had a poorer age-specific body condition^9^. Similar negative effects have been found among rodents live-trapped over two years^15^. Neither tail fat index nor body length were affected short or long-term by repeated captures and handling in our population. However, we did find a clear decrease in body mass with increasing capture and handling events among dominant individuals. Other studies in beavers have observed greater weight loss and decrease in tail area over the following winter when being equipped with transmitters^16^. We might expect more short-term effects within days post-capture related to the individuals staying more in lodge and being less active as an immediate response to the capture event^49^.

Tail fat index, body mass and body size increased considerably between life stages of beavers, reflecting their growth rate. Furthermore, they increased over the year, especially among young individuals, which increase relatively more in mass and size. But also adults showed seasonal variations similar with findings in other populations^56^.

Females in the reproductive age generally had smaller tail size and higher body mass in spring and summer when reproduction occurs, indicating differentiation in allocation of time and energy among sexes. Comparing time-budgets of males and females, no differences were found^46^, which is expected in monogamous animals^57^. Males allocated more time to travel which might indicate an increased patrolling effort or search for food items, whereas females might spend more time on parenting activities, which our results support.

Only body mass was greater among dominant individuals, indicating this as an important feature of social status^58^, whereas tail size and body length might be more socially stable to maximize fitness of individuals^37^. Social rank is highly shaped by agonistic interactions between individuals^59^, where body size can be an important factor^60^. However, the social hierarchy may also be influenced by affiliative interactions^61^. In beavers only the dominating pair breed. Also among high ranked individuals in other social rodents have body size correlated with greater reproductive success^62^. In our population, body mass decreased among dominant individuals with increasing number of capture and handling events. Dominants have a higher energy expenditure due to increased territorial defence and reproduction^36,37,41^, which may suggest they might be more susceptible to capture stress^7,60^.

Young individuals of larger territories were found to have lower body mass and smaller body length, which might be a result of parents spending more time on patrolling than parenting^47^. Generally, costs of defending a territory are positively correlated with territory size^63^ and young individuals might suffer as a consequence. Larger territories may have fewer quality food resources and might be larger to provide adequate resource availability^64–66^. However, we found no effect of territory size among adults suggesting that territorial behaviour in our population, to a lesser degree, follows the availability of food resources^67^.

### Reproduction

Capture and handling may have further deleterious effects on individuals beyond the immediate event of capture, potentially affecting the following breeding season, but may also have long-term consequences for the reproductive success of handled individuals^20^. We did not find any statistically clear effects of repeated captures and handling on the annual probability of reproducing in our population, which is consistent with results from other long-term studies^6,68^. Studies in ungulates lasting up to 15 years found no evidence of lowered calving success among immobilized individuals^8,20^. However, we did find a decrease in annual litter size with increasing capture and handling events among older individuals. Other studies lasting up to 30 years in ungulates, birds, bears, as well as other species, have similarly showed negative effects on breeding success of repeated capture and handling^17,19,24,52,69,70^. Even though several studies investigated capture and handling effects over several years and seasons, most focus on effects in the current or subsequent breeding season, only assessing short-term effects in species that goes through several seasons. However, capture and handling might induce long-term effects, influencing reproduction in future life-history stages^4,71^. Not evaluating the long-term effects, we risk overlooking important fitness effects^9,52^.

In our study, we found varying capture effects on the reproductive success over more than 20 years of monitoring. Repeated capture and handling had strong negative effects on annual reproduction in the early years of monitoring, which decreased considerably in recent years, indicating habituation to capture and handling stress^32,72^. To our knowledge, similar reproduction responses to repeated capture events have not been observed in other long-term studies, but habituation responses to handling and human activities have been found for behaviour related to breeding in shorebirds^73^ and spatial behaviour and alertness in birds and mammals^74–79^. Some behaviour might even be shaped by parental habituation^34^. Additionally, habituation might be affected by the number of experimenters^80^, requiring thoughtful planning of experimental setups to minimize observational effects.

### Survival

Mortality rates and physical injuries at time of capture are not sufficient to successfully assess capture and handling effects, as animals often hide symptoms to avoid further harm^10,11^. Several studies have found negative effects of capture and handling on survival in birds and mammals in the weeks after capture^5,21,23,26,27,29^ while others observe no effects^19,22,28,51,81^, but most studies do not address long-term effects. A seven year penguin study found decreased annual survival^25^, but survival varied considerably between years, underlining the challenge of finding clear capture and handling effect on survival of individuals^10^. We found no statistically clear short or long-term capture effects on survival in our population. Similar results were found comparing the effects of transmitters over eight years in beavers^16^. However, more details on actual time of death of individuals may reveal capture and handling-related effects that otherwise may go undetected^9,52^.

Social status had a strong impact on annual survival in our population and handling procedures that affect the possibility to obtain or hold a social rank might have considerable consequences^7^. Subordinate individuals may be more challenged than territory holders to find a territory of their own, which may result in lower survival or accepting a territory of lesser quality as seen in other rodents^82^. Being potentially physiologically pressured (e.g. from growing) and operating in a highly tense social landscape^83^, we would expect high capture intensity in combination to further affect survival^7^, whereas dominant individuals might be more socially anchored in a territory and therefore more resilient^82^. We found a statistically clear decrease in survival of subordinates with increasing age and with increasing family group size which indicate they might be better off leaving their family group after some time. Subordinate beavers have been shown to make explorative trips to neighbouring and distant territories before they disperse from their natal family group^84^ and might experience increased mortality risk when exploring potential territories^85–87^.

Dominant individuals might need time to establish in a territory and build up their family group. Accordingly, we found a higher probability of staying dominant for individuals in larger family groups. A high number of individuals in a territory might indicate high territory quality^88^ which the prolonged territory occupancy might reflect. Furthermore, the relationship between larger family groups and territory occupancy might reflect the benefits of social behaviour in beavers^89^. Other rodent studies found an increased survival and reproductive success among individuals that were translocated to new territories together with familiar conspecifics^90,91^. Not surprisingly, the probability of keeping dominance decreased with increasing age, indicating senescence^83,92^.

## Conclusion

Using a semi-aquatic mammal as a model species, we here present clear insights on how long-term monitoring studies including repeated capturing and handling of individuals affect the wellbeing of wild animal populations by evaluating changes in several fitness-related parameters. Our results, based on more than 20 years of a unique long-term individual-based field study, show how repeated capture and handling can have clear effects on important life stages in a semi-aquatic mammal. We illustrate the validity of long-term individual-based studies which have great potential in the planning and execution of future wildlife and conservation studies to promote the welfare of wild populations and ensure reliable research data^12^.

Long-term individual-based studies should always evaluate the potential bias created. Our results confirm the importance of having a clear adaptive objective when assessing effects of repeated capture and handling. Setting clear objectives and framing tractable questions will help resolve on what to monitor and assess, allowing the monitoring program to evolve and develop in response to new information and new questions^93^.

## Methods

### Study animal

Beavers are socially monogamous, semi-aquatic nocturnal mammals that inhabit various freshwater bodies^42^. They live in family groups consisting of the dominant breeding pair, kits of the year and older non-breeding offspring^94^. Beavers reach sexual maturity during their second winter^37^ and give birth to one to five kits in mid-May, which emerge from the lodge during July when they start feeding on their own on twigs and leaves of deciduous trees like the adults^94^. Around 2–3 years old, beavers disperse to establish a territory of their own^84,95^.

Beavers are highly territorial and announce territory occupation mainly by scent marking, primarily at territorial borders^39^. All adults take part in territorial defence, but males allocate more time to patrolling and scent marking^46^.

### Study site

Our study site is located at the lower reaches of the rivers Straumen, Gvarv and Sauar in Vestfold and Telemark County, south-eastern Norway. The river sections are generally slow flowing with stable water levels, have similar depth structure and are 20–150 m wide^96^. All rivers contain natural lakes and man-made impoundments along part of their length, resulting in only limited water temperature fluctuations along the main river channels^96^. The rivers flow through small towns, farms and fields interspersed with riparian woodland^44,45^.

Beavers have inhabited the rivers since the 1920s where they recolonized the rivers. The population is at carrying capacity, as territories of various sizes border each other directly^94^. Territory borders are identified based on scent mound concentrations, sight observations of patrolling known beavers and GPS data.

### Capture protocol

Since 1997, beavers in the area have been monitored through an extensive live-trapping program (the Norwegian Beaver Project, NBP). The long-term monitoring project aims to annually capture all newcomers (kits and dispersers from outside the study site) to enable identification at later encounters, as well as annually record family group sizes.

Captures were conducted at night. Individuals were detected from a motorboat using searchlights and captured using large landing-nets in shallow water or on land^97^. Captured individuals were immobilized in cloth sacks, enabling easy handling without anaesthesia. Beavers were weighed to the nearest 100 g. Body length was measured following the curvature of the spine from nose tip to the base of the tail. Tail length was measured from the base to the tip of the tail and tail width was measured from edge to edge of the dorsal surface at the midpoint between tail base and tip.

When encountering individuals, samples of castoreum and anal gland secretion were obtained. Hair samples from the lower back were obtained at an individual’s first encounter. Furthermore, some adults participated in short-term experiments involving deployment of various data-loggers and transmitters. Unfamiliar individuals were sexed based on the colour of their anal gland secretion^98^, and tagged with microchips and unique combinations of plastic and metal ear-tags. Individuals first captured as kit or yearling were given an exact age. Older individuals (≥ 2 years) were assigned a minimum age based on body mass when first captured^99^; minimum 2 years (subadult) when ≥ 17 and ≤ 19.5 kg and minimum 3 years (adult) when > 19.5 kg. Dominance was in most cases attributed to adult territorial residents of each sex. Dominance was otherwise verified by eventual dispersal of the alternative candidate, greatest body weight among same-sex group members or lactation in females (large nipples). Individuals dispersing into a territory were posited to have achieved the dominant breeding position when the previous dominant of the same sex had disappeared or evidence outlined above were applicable. Unless proven otherwise, dominant individuals were assumed to maintain their status until they disappeared or died^100^. Captured beavers were released near capture site within their territory after 20–40 min of handling time^97^.

### Ethical note

All capture and handling procedures were approved by the Norwegian Experimental Animal Board (Most recent authorization: FOTS ID15947) and by the Norwegian Directorate for Nature Management (Most recent authorization: 2014/14415). Our study met the ASAB/ABS Guidelines for the treatment of animals in behavioural research and teaching^101^. No captured individuals were injured during capture and handling, and all were successfully released afterwards. All methods were performed in accordance with the relevant guidelines and regulations.

### Body condition indices

The beaver tail functions as fat storage^102^. Tail size (length × width) varies with season and is positively correlated with body mass^56^. Using the ratio of the tail size (seasonally variable) to body length (seasonally stable) as an index of tail fat content, tail size has been used as proxy for body condition^37,103^. Larger tail fat index thus indicates higher tail fat content and better body condition.

However, body mass and body length may vary differently than tail fat in relation to long-term capture and handling effects. Body length was not recorded in a few capturing events (21 individuals), but tail size was measured. In those cases, we interpolated body length between measures within the individual in an age group (i.e., kit, yearling, subadult and adult) and excluded observations if interpolation was not possible within the age group.

Since young and adults are not treated equally in our protocol and may be affected by different ecological factors, we chose to divide our analyses on body condition indices into young (kits and yearlings) and adults (subadults and adults) to balance the data set.

### Reproduction

Each year we aimed to capture all kits and yearlings not captured during their first year. We recorded annual reproductive success for breeding pairs in each territory as the number of kits in a given year, based on the number of captured and observed kits plus unmarked yearlings captured the following year.

### Survival

Using observations from the capture protocol in a capture-mark-recapture (CMR) framework, we can estimate apparent survival in the population, which may reflect actual survival, but also emigration from the study area^104–106^.

As dispersal is a dangerous period during the life of an animal, which may result in high mortality^107,108^, territory occupancy of dominant individuals may be used as proxy for survival assuming dominant individuals perished when they lost territory occupancy and did not overtake a new territory^83^.

### Statistical analysis

For all analyses on body condition, reproduction and dominance status we used linear mixed-effects models (LMM) and generalized linear mixed-effects models (GLMM) with capture year, beaver ID and study river as random effects to account for variability between years, individuals and study sites that might be caused by climatic differences, habitat quality or sampling frequency.

We investigated how capture and handling affected tail fat index, body mass and body length using LMMs for young and adult individuals, respectively. For young individuals we analysed how the number of capture and handling events and years of monitoring (years since 1997) affected the tail fat index, body mass and body length of individuals. Capture season (spring: March–May, summer: June–August, autumn: September–November), sex, age, territory size (km bank length) and family group size were included to account for important ecological factors. Similar LMMs were built for adults, additionally analysing the effect of carrying a telemetry device (yes/no) in its lifetime up to the given capture event, accounting for social status of the beaver (dominant/subordinate) at the capture event and the individual’s family origin (immigrant or resident).

To investigate effects of long-term capture and handling on annual reproduction, we used a GLMM with binomial distribution and logit link (1 = reproducing dominant females, 0 = non-reproducing dominant females), analysing the relative importance of number of capture and handling events, years of monitoring and whether the animal had carried a telemetry device in its life time. We included age of the beaver, whether the female reproduced the previous year, territory size family origin and size of family group. The same covariates were included in a GLMM with Poisson distribution and log link to analyse the effect of long-term captures and handling on annual reproductive success (i.e. litter size).

To investigate effects of long-term capture and handling on dominance status, we used a GLMM with binomial distribution and logit link (1 = individuals that kept dominance in the following year, 0 = individuals that lost dominance in the following year), analysing the relative importance of number of capture and handling events, years of monitoring and whether the animal had carried a telemetry device in its life time for keeping dominance status the following year, including effects of sex, age, territory size, family origin and family group size.

The fixed effects used in all analyses were not correlated (Pearson r coefficient < 0.6) and variance inflation factor values were < 3^109^.

A list of candidate models was created using ecologically relevant combinations of fixed effects. Variables were included to account for variability in endogenous (such as sex, age and social status) and exogenous factors (such as territory size, family group size, family origin and season) that are important for describing the ecology of the beaver. Years since 1997 (years of monitoring) were included to capture the long-term effects of our capture protocol, as the monitoring may not only affect individuals that are monitored at a given time but may also affect future generations. Years of monitoring for a given individual at a given time is implicitly within the age of the beaver (r = 0.9) and in the number of captures (r = 0.7). Linearity of variables were tested in univariable mixed-effect models with either linear or squared variables. We included interactions to capture the variability in how the beavers reacts to capture and handling events on a long term (Captures × Years of monitoring), and the variability in how males, females, dominants and subordinates, young and old may react to repeated capture and handling events (Captures × Sex, Captures × Social status, Capture × Age, respectively). Furthermore, we included interactions to account for ecological differences between males and females over the year (Season × Sex) and the variability for social status at different ages (Social status × Age), as there is a higher degree of subordinates among young individuals and a higher degree of dominants among older individuals.

Model selection was based on Akaike’s Information Criterion corrected for small sample size^110^, and carried out using the R packages glmmTMB^111^ and MuMIn^112^. If ΔAICc was < 4 in two or more of the most parsimonious models, we performed model averaging^110,113^. Parameters that included zero within their 95% confidence interval (CI) were considered uninformative^113^. The most parsimonious models were visually validated using the R package DHARMa^114^ to plot standardised model residuals against the fitted values^109^. Models for reproduction and dominance status were furthermore checked for zero-inflation using DHARMa. Top candidate models for all analyses can be found in the supporting material.

Survival probability was modeled in a CMR framework. Since classical CMR models require discrete-time assumptions^106^ that introduce constraints in CMR protocols that are not always compatible with the reality^104,105^, we chose to fit our survival model in a continuous time capture-recapture model using the R-package CMRCT^104^, enabling us to estimate the apparent annual survival in our population. We modeled how the importance of repeated capture and handling events, years of monitoring and carrying a telemetry device in its lifetime affected apparent survival. Capture season, sex, age, territory size and family group size were included to account for important ecological factors. Furthermore we included interactions described above (Supplementary information S1).

As CMRCT currently does not offer any model selection, we fitted all variables and interactions in a global model and removed uninformative parameters by backwards selection until the model consisted only of informative parameters. Parameters that included zero within their 95% confidence interval (CI) were considered uninformative^113^.

All analyses were conducted in R 3.6.3^115^.

## Supplementary information

Supplementary Information

## Data Availability

Data is available at 10.23642/usn.13083782.
