# Supplementary material for: Long-term capture and handling effects on body condition, reproduction and survival in a semi-aquatic mammal
Source: Sci Rep. 2020 Oct 21;10:17886. doi: 10.1038/s41598-020-74933-w (PMC7578049; doi:10.1038/s41598-020-74933-w)
Supplement: Supplementary file 1 — Supplementary Information [file 41598_2020_74933_MOESM1_ESM.docx]

**Long-term capture and handling effects on body condition, reproduction and survival in a semi-aquatic mammal**

Rasmus M. Mortensen^1*^ and Frank Rosell^1^

^1^ Faculty of Technology, Natural Sciences, and Maritime Sciences, Department of Natural Sciences and Environmental Health, University of South-Eastern Norway, Bø i Telemark, Norway

^*^ Corresponding author: [rmo@usn.no](mailto:rmo@usn.no)

**Supplemental material**

**S1.** The model selection result for the candidate models investigating the tail fat index in young individuals in a Eurasian beaver population in south-eastern Norway between 1998 and 2019. Beaver ID, capture year and river were included as random effects. Models were ranked based on AICc.

| Variables | df | log likelihood | AICc | deltaAICc | weight | **R^2^_marginal_** | **R^2^_conditional_** |
| --- | --- | --- | --- | --- | --- | --- | --- |
| 1/4/6 | 9 | -153.26 | 325.08 | 0.00 | 0.18 | 0.56 | 0.73 |
| 1/4/5/6 | 10 | -152.69 | 326.05 | 0.97 | 0.11 | 0.56 | 0.73 |
| 1/4/6/7 | 10 | -152.77 | 326.22 | 1.14 | 0.10 | 0.56 | 0.73 |
| 1/3/4/6 | 10 | -152.88 | 326.45 | 1.37 | 0.09 | 0.56 | 0.74 |
| 1/3/4/5/6 | 11 | -152.18 | 327.19 | 2.10 | 0.06 | 0.56 | 0.74 |
| 1/2/4/6 | 10 | -153.26 | 327.20 | 2.12 | 0.06 | 0.56 | 0.73 |
| 1/4/5/6/7 | 11 | -152.23 | 327.27 | 2.19 | 0.06 | 0.56 | 0.73 |
| 1/3/4/6/7 | 11 | -152.41 | 327.65 | 2.57 | 0.05 | 0.56 | 0.74 |
| 1/4 | 8 | -155.65 | 327.75 | 2.67 | 0.05 | 0.55 | 0.73 |
| 1/2/4/5/6 | 11 | -152.68 | 328.19 | 3.11 | 0.04 | 0.56 | 0.73 |
| 1/4/5 | 9 | -154.85 | 328.26 | 3.18 | 0.04 | 0.56 | 0.73 |
| 1/2/4/6/7 | 11 | -152.76 | 328.35 | 3.26 | 0.04 | 0.56 | 0.73 |
| 1/3/4/5/6/7 | 12 | -151.75 | 328.47 | 3.39 | 0.03 | 0.56 | 0.74 |
| 1/2/3/4/6 | 11 | -152.85 | 328.51 | 3.43 | 0.03 | 0.56 | 0.74 |
| 1/4/7 | 9 | -155.17 | 328.90 | 3.82 | 0.03 | 0.55 | 0.73 |
| 1/3/4 | 9 | -155.17 | 328.90 | 3.82 | 0.03 | 0.55 | 0.74 |
| 1 = Age, 2 = Captures, 3 = Family group size, 4 = Season, 5 = Sex, 6 = log(Territory size), 7 = Years of monitoring | | | | | |  |  |

**S2.** The model selection result for the candidate models investigating the tail fat index in adult individuals in a Eurasian beaver population in south-eastern Norway between 1998 and 2019. Beaver ID, capture year and river were included as random effects. Models were ranked based on AICc.

| Variables | df | log likelihood | AICc | deltaAICc | weight | **R^2^_marginal_** | **R^2^_conditional_** |
| --- | --- | --- | --- | --- | --- | --- | --- |
| 1/2/4/8/9/12/14 | 15 | -229.32 | 489.24 | 0.00 | 0.04 | 0.12 | 0.48 |
| 1/2/4/8/9/12 | 13 | -231.44 | 489.32 | 0.08 | 0.04 | 0.12 | 0.48 |
| 1/4/8/9/12/14 | 14 | -230.63 | 489.78 | 0.54 | 0.03 | 0.12 | 0.48 |
| 1/4/8/9/12 | 12 | -232.79 | 489.95 | 0.72 | 0.03 | 0.11 | 0.48 |
| 1/2/4/8/9/10/12/14 | 16 | -228.88 | 490.44 | 1.20 | 0.02 | 0.13 | 0.48 |
| 1/3/4/8/9/12 | 13 | -232.05 | 490.54 | 1.30 | 0.02 | 0.12 | 0.48 |
| 1/2/4/8/9/10/12 | 14 | -231.03 | 490.58 | 1.34 | 0.02 | 0.12 | 0.47 |
| 1/3/4/8/9/12/14 | 15 | -230.03 | 490.65 | 1.41 | 0.02 | 0.12 | 0.48 |
| 1/4/8/9/10/12/14 | 15 | -230.06 | 490.70 | 1.46 | 0.02 | 0.12 | 0.48 |
| 1/2/4/5/8/9/12/14 | 16 | -229.05 | 490.78 | 1.54 | 0.02 | 0.13 | 0.48 |
| 1/2/4/5/8/9/12 | 14 | -231.17 | 490.85 | 1.61 | 0.02 | 0.12 | 0.48 |
| 1/4/8/9/10/12 | 13 | -232.25 | 490.94 | 1.71 | 0.02 | 0.12 | 0.48 |
| 1/2/4/8/9/11/12 | 14 | -231.29 | 491.10 | 1.86 | 0.02 | 0.12 | 0.48 |
| 1/4/8/9/11/12/14 | 15 | -230.26 | 491.12 | 1.88 | 0.02 | 0.12 | 0.48 |
| 1/2/3/4/8/9/12 | 14 | -231.33 | 491.18 | 1.94 | 0.02 | 0.12 | 0.48 |
| 1/2/3/4/8/9/12/14 | 16 | -229.27 | 491.21 | 1.97 | 0.02 | 0.12 | 0.48 |
| 1/2/4/8/9/12/13/14 | 16 | -229.27 | 491.21 | 1.97 | 0.02 | 0.12 | 0.48 |
| 1/2/8/9/14 | 13 | -232.41 | 491.26 | 2.03 | 0.02 | 0.12 | 0.48 |
| 1/2/4/7/8/9/12/14 | 16 | -229.30 | 491.27 | 2.03 | 0.02 | 0.12 | 0.48 |
| 1/2/4/8/9/12/13 | 14 | -231.41 | 491.33 | 2.09 | 0.02 | 0.12 | 0.48 |
| 1/4/8/9/11/12 | 13 | -232.45 | 491.35 | 2.11 | 0.02 | 0.12 | 0.48 |
| 1/2/4/7/8/9/12 | 14 | -231.42 | 491.36 | 2.12 | 0.02 | 0.12 | 0.48 |
| 1/2/8/9 | 11 | -234.57 | 491.47 | 2.23 | 0.01 | 0.12 | 0.47 |
| 1/4/5/8/9/12/14 | 15 | -230.44 | 491.47 | 2.23 | 0.01 | 0.12 | 0.49 |
| 1/3/4/8/9/10/12 | 14 | -231.53 | 491.58 | 2.34 | 0.01 | 0.12 | 0.47 |
| 1/4/7/8/9/12/14 | 15 | -230.50 | 491.59 | 2.36 | 0.01 | 0.12 | 0.49 |
| 1/3/4/8/9/10/12/14 | 16 | -229.47 | 491.62 | 2.38 | 0.01 | 0.12 | 0.48 |
| 1/4/5/8/9/12 | 13 | -232.60 | 491.64 | 2.40 | 0.01 | 0.12 | 0.48 |
| 1/4/7/8/9/12 | 13 | -232.62 | 491.70 | 2.46 | 0.01 | 0.12 | 0.48 |
| 1/2/4/5/8/9/10/12/14 | 17 | -228.50 | 491.76 | 2.52 | 0.01 | 0.13 | 0.48 |
| 1/2/4/8/9 | 12 | -233.69 | 491.76 | 2.52 | 0.01 | 0.12 | 0.47 |
| 1/2/4/8/9/13/14 | 15 | -230.65 | 491.89 | 2.65 | 0.01 | 0.12 | 0.48 |
| 1/2/4/5/8/9/10/12 | 15 | -230.66 | 491.90 | 2.66 | 0.01 | 0.13 | 0.47 |
| 1/4/8/9/10/11/12/14 | 16 | -229.71 | 492.09 | 2.85 | 0.01 | 0.12 | 0.48 |
| 1/3/4/5/8/9/12 | 14 | -231.82 | 492.16 | 2.92 | 0.01 | 0.12 | 0.48 |
| 1/4/5/8/9/10/12/14 | 16 | -229.75 | 492.18 | 2.94 | 0.01 | 0.12 | 0.48 |
| 1/2/4/8/9/10/11/12/14 | 17 | -228.72 | 492.19 | 2.95 | 0.01 | 0.13 | 0.48 |
| 1/3/4/8/9/11/12 | 14 | -231.87 | 492.25 | 3.01 | 0.01 | 0.12 | 0.48 |
| 1/3/4/5/8/9/12/14 | 16 | -229.80 | 492.28 | 3.04 | 0.01 | 0.12 | 0.48 |
| 1/3/4/8/9/11/12/14 | 16 | -229.81 | 492.29 | 3.05 | 0.01 | 0.12 | 0.48 |
| 1/2/4/8/9/13 | 13 | -232.94 | 492.33 | 3.10 | 0.01 | 0.12 | 0.48 |
| 1/2/8/9/10/14 | 14 | -231.93 | 492.37 | 3.13 | 0.01 | 0.12 | 0.47 |
| 1/4/8/9/10/11/12 | 14 | -231.93 | 492.38 | 3.15 | 0.01 | 0.12 | 0.48 |
| 1/2/3/4/8/9/10/12/14 | 17 | -228.82 | 492.39 | 3.15 | 0.01 | 0.13 | 0.48 |
| 1/2/3/4/8/9/10/12 | 15 | -230.91 | 492.42 | 3.18 | 0.01 | 0.12 | 0.47 |
| 1/4/5/8/9/10/12 | 14 | -231.95 | 492.42 | 3.19 | 0.01 | 0.12 | 0.48 |
| 1/2/4/8/9/10/12/13/14 | 17 | -228.84 | 492.43 | 3.19 | 0.01 | 0.13 | 0.48 |
| 1/2/4/7/8/9/10/12/14 | 17 | -228.85 | 492.46 | 3.22 | 0.01 | 0.13 | 0.48 |
| 1/2/4/8/9/10/14 | 15 | -230.95 | 492.49 | 3.25 | 0.01 | 0.12 | 0.47 |
| 1/3/4/7/8/9/12 | 14 | -231.99 | 492.51 | 3.27 | 0.01 | 0.12 | 0.48 |
| 1/4/8/9/14 | 13 | -233.05 | 492.54 | 3.30 | 0.01 | 0.11 | 0.48 |
| 1/8/9/14 | 12 | -234.08 | 492.55 | 3.31 | 0.01 | 0.11 | 0.48 |
| 1/4/7/8/9/10/12/14 | 16 | -229.96 | 492.58 | 3.34 | 0.01 | 0.12 | 0.48 |
| 1/2/4/8/9/10/12/13 | 15 | -231.01 | 492.61 | 3.37 | 0.01 | 0.12 | 0.47 |
| 1/2/4/7/8/9/10/12 | 15 | -231.01 | 492.62 | 3.38 | 0.01 | 0.12 | 0.47 |
| 1/2/8/9/10 | 12 | -234.13 | 492.63 | 3.40 | 0.01 | 0.12 | 0.47 |
| 1/3/4/7/8/9/12/14 | 16 | -229.99 | 492.65 | 3.41 | 0.01 | 0.12 | 0.48 |
| 1/2/3/4/5/8/9/12 | 15 | -231.06 | 492.70 | 3.47 | 0.01 | 0.12 | 0.48 |
| 1/2/3/4/5/8/9/12/14 | 17 | -228.99 | 492.74 | 3.50 | 0.01 | 0.13 | 0.48 |
| 1/4/7/8/9/10/12 | 14 | -232.12 | 492.76 | 3.52 | 0.01 | 0.12 | 0.48 |
| 1/4/5/8/9/11/12/14 | 16 | -230.05 | 492.77 | 3.53 | 0.01 | 0.12 | 0.49 |
| 1/2/4/5/7/8/9/12/14 | 17 | -229.03 | 492.81 | 3.57 | 0.01 | 0.13 | 0.48 |
| 1/2/4/5/8/9/12/13/14 | 17 | -229.03 | 492.82 | 3.58 | 0.01 | 0.13 | 0.48 |
| 1/8/9 | 10 | -236.30 | 492.87 | 3.63 | 0.01 | 0.11 | 0.47 |
| 1/2/4/5/7/8/9/12 | 15 | -231.15 | 492.89 | 3.65 | 0.01 | 0.12 | 0.48 |
| 1/2/4/5/8/9/12/13 | 15 | -231.16 | 492.91 | 3.67 | 0.01 | 0.12 | 0.48 |
| 1/3/4/5/8/9/10/12 | 15 | -231.19 | 492.97 | 3.73 | 0.01 | 0.12 | 0.47 |
| 1/4/8/9 | 11 | -235.33 | 492.99 | 3.75 | 0.01 | 0.11 | 0.48 |
| 1/4/7/8/9/11/12/14 | 16 | -230.16 | 493.00 | 3.76 | 0.01 | 0.12 | 0.49 |
| 1/4/5/8/9/11/12 | 14 | -232.24 | 493.00 | 3.76 | 0.01 | 0.12 | 0.48 |
| 1/3/4/5/8/9/10/12/14 | 17 | -229.13 | 493.01 | 3.77 | 0.01 | 0.12 | 0.48 |
| 1/2/3/4/8/9/11/12 | 15 | -231.21 | 493.02 | 3.78 | 0.01 | 0.12 | 0.48 |
| 1/2/4/7/8/9/11/12/14 | 17 | -229.13 | 493.02 | 3.78 | 0.01 | 0.13 | 0.48 |
| 1/2/4/8/9/10/13/14 | 16 | -230.19 | 493.05 | 3.81 | 0.01 | 0.12 | 0.48 |
| 1/2/4/8/9/11/12/13 | 15 | -231.26 | 493.11 | 3.87 | 0.01 | 0.12 | 0.48 |
| 1/2/4/8/9/10/11/12 | 15 | -231.27 | 493.14 | 3.90 | 0.01 | 0.13 | 0.47 |
| 1/2/8/9/11/14 | 14 | -232.32 | 493.15 | 3.91 | 0.01 | 0.12 | 0.48 |
| 1/4/7/8/9/11/12 | 14 | -232.32 | 493.16 | 3.92 | 0.01 | 0.12 | 0.48 |
| 1/2/3/4/8/9/12/13/14 | 17 | -229.22 | 493.19 | 3.95 | 0.01 | 0.12 | 0.48 |
| 1/2/3/4/8/9/12/13 | 15 | -231.31 | 493.20 | 3.96 | 0.01 | 0.12 | 0.48 |
| 1/2/3/4/7/8/9/12 | 15 | -231.32 | 493.23 | 3.99 | 0.01 | 0.12 | 0.48 |
| 1 = Age+Age^2^, 2 = Captures, 3 = Carried telemetry device, 4 = Social status, 5 = Family group size, 7 = Origin, 8 = Season, 9 = Sex, 10 = log(Territory size), 11 = Years of monitoring, 12 = Social status:Age, 13 = Captures:Social status, 14 = Season:Sex | | | | | |  |  |

**S3.** The model selection result for the candidate models investigating the body mass in young individuals in a Eurasian beaver population in south-eastern Norway between 1998 and 2019. Beaver ID, capture year and river were included as random effects. Models were ranked based on AICc.

| Variables | df | log likelihood | AICc | deltaAICc | weight | **R^2^_marginal_** | **R^2^_conditional_** |
| --- | --- | --- | --- | --- | --- | --- | --- |
| 1/2/3/4/6 | 11 | -561.97 | 1146.75 | 0.00 | 0.20 | 0.87 | 0.93 |
| 1/3/4/6/ | 10 | -563.12 | 1146.92 | 0.17 | 0.18 | 0.87 | 0.93 |
| 1/2/3/4/6/7 | 12 | -561.24 | 1147.43 | 0.68 | 0.14 | 0.87 | 0.93 |
| 1/3/4/6/7 | 11 | -562.42 | 1147.64 | 0.89 | 0.13 | 0.87 | 0.93 |
| 1/2/3/4/5/6 | 12 | -561.93 | 1148.81 | 2.06 | 0.07 | 0.87 | 0.93 |
| 1/3/4/5/6 | 11 | -563.08 | 1148.97 | 2.22 | 0.07 | 0.87 | 0.93 |
| 1/2/3/4/5/6/7 | 13 | -561.20 | 1149.52 | 2.77 | 0.05 | 0.87 | 0.93 |
| 1/3/4/5/6/7 | 12 | -562.39 | 1149.72 | 2.98 | 0.05 | 0.87 | 0.93 |
| 1/4/6 | 9 | -565.60 | 1149.75 | 3.00 | 0.04 | 0.87 | 0.93 |
| 1/4/6/7 | 10 | -564.81 | 1150.29 | 3.54 | 0.03 | 0.87 | 0.93 |
| 1/2/4/6 | 10 | -564.99 | 1150.65 | 3.90 | 0.03 | 0.87 | 0.93 |
| 1 = Age, 2 = Captures, 3 = Family group size, 4 = Season, 5 = Sex, 6 = log(Territory size), 7 = Years of monitoring | | | | | |  |  |

**S4.** The model selection result for the candidate models investigating the body mass in adult individuals in a Eurasian beaver population in south-eastern Norway between 1998 and 2019. Beaver ID, capture year and river were included as random effects. Models were ranked based on AICc.

| Variables | df | log likelihood | AICc | deltaAICc | weight | **R^2^_marginal_** | **R^2^_conditional_** |
| --- | --- | --- | --- | --- | --- | --- | --- |
| 1/2/4/5/8/9/11/12/14/15/16 | 19 | -1854.13 | 3747.10 | 0.00 | 0.22 | 0.48 | 0.78 |
| 1/2/4/5/8/9/11/12/13/14/15/16 | 20 | -1853.94 | 3748.82 | 1.72 | 0.09 | 0.48 | 0.78 |
| 1/2/4/5/8/9/10/11/12/14/15/16 | 20 | -1854.10 | 3749.13 | 2.03 | 0.08 | 0.48 | 0.78 |
| 1/2/4/8/9/11/12/14/15/16 | 18 | -1856.20 | 3749.16 | 2.06 | 0.08 | 0.48 | 0.78 |
| 1/2/3/4/5/8/9/11/12/14/15/16 | 20 | -1854.12 | 3749.17 | 2.07 | 0.08 | 0.48 | 0.78 |
| 1/2/4/5/7/8/9/11/12/14/15/16 | 20 | -1854.13 | 3749.19 | 2.09 | 0.08 | 0.48 | 0.78 |
| 1/2/4/8/9/11/12/13/14/15/16 | 19 | -1855.69 | 3750.23 | 3.13 | 0.05 | 0.48 | 0.78 |
| 1/2/4/5/8/9/12/14/16 | 17 | -1857.83 | 3750.34 | 3.24 | 0.04 | 0.47 | 0.78 |
| 1/2/4/5/8/9/11/12/14/15 | 17 | -1857.92 | 3750.53 | 3.43 | 0.04 | 0.48 | 0.78 |
| 1/2/4/5/8/9/14/16 | 16 | -1858.97 | 3750.54 | 3.44 | 0.04 | 0.48 | 0.78 |
| 1/2/4/5/8/9/10/11/12/13/14/15/16 | 21 | -1853.91 | 3750.85 | 3.75 | 0.03 | 0.48 | 0.78 |
| 1/2/4/5/8/9/11/12/14/16 | 18 | -1857.06 | 3750.89 | 3.79 | 0.03 | 0.48 | 0.78 |
| 1/2/3/4/5/8/9/11/12/13/14/15/16 | 21 | -1853.94 | 3750.91 | 3.81 | 0.03 | 0.48 | 0.78 |
| 1/2/4/5/7/8/9/11/12/13/14/15/16 | 21 | -1853.94 | 3750.91 | 3.81 | 0.03 | 0.48 | 0.78 |
| 1/2/4/5/8/9/11/14/15/16 | 18 | -1857.09 | 3750.95 | 3.85 | 0.03 | 0.49 | 0.78 |
| 1/2/4/8/9/10/11/12/14/15/16 | 19 | -1856.10 | 3751.05 | 3.95 | 0.03 | 0.48 | 0.78 |
| 1 = Age+Age^2^, 2 = Captures, 3 = Carried telemetry device, 4 = Social status, 5 = Family group size, 7 = Origin, 8 = Season, 9 = Sex, 10 = log(Territory size), 11 = Years of monitoring, 12 = Captures:Age, 13 = Social status:Age, 14 = Captures:Social status, 15 = Captures:Years of monitoring, 16 = Season sex | | | | | |  |  |

**S5.** The model selection result for the candidate models investigating the body size in young individuals in a Eurasian beaver population in south-eastern Norway between 1998 and 2019. Beaver ID, capture year and river were included as random effects. Models were ranked based on AICc.

| Variables | df | log likelihood | AICc | deltaAICc | weight | **R^2^_marginal_** | **R^2^_conditional_** |
| --- | --- | --- | --- | --- | --- | --- | --- |
| 1/3/4/6 | 10 | -964.75 | 1950.19 | 0.00 | 0.17 | 0.78 | 0.84 |
| 1/4/6/7 | 10 | -964.81 | 1950.30 | 0.11 | 0.16 | 0.78 | 0.83 |
| 1/3/4/6/7 | 11 | -963.87 | 1950.57 | 0.38 | 0.14 | 0.78 | 0.84 |
| 1/4/5/6 | 10 | -965.55 | 1951.79 | 1.60 | 0.08 | 0.77 | 0.84 |
| 1/2/4/6 | 10 | -965.65 | 1951.99 | 1.80 | 0.07 | 0.77 | 0.84 |
| 1/4/5/6/7 | 11 | -964.69 | 1952.21 | 2.02 | 0.06 | 0.78 | 0.83 |
| 1/2/3/4/6 | 11 | -964.70 | 1952.23 | 2.04 | 0.06 | 0.78 | 0.84 |
| 1/3/4/5/6 | 11 | -964.71 | 1952.23 | 2.05 | 0.06 | 0.78 | 0.84 |
| 1/2/4/6/7 | 11 | -964.80 | 1952.42 | 2.24 | 0.06 | 0.78 | 0.83 |
| 1/2/3/4/6/7 | 12 | -963.81 | 1952.60 | 2.41 | 0.05 | 0.78 | 0.84 |
| 1/3/4/5/6/7 | 12 | -963.82 | 1952.61 | 2.43 | 0.05 | 0.78 | 0.84 |
| 1/2/4/5/6 | 11 | -965.55 | 1953.93 | 3.74 | 0.03 | 0.77 | 0.84 |
| 1 = Age, 2 = Captures, 3 = Family group size, 4 = Season, 5 = Sex, 6 = log(Territory size), 7 = Years of monitoring | | | | | |  |  |

**S6.** The model selection result for the candidate models investigating the body size in adult individuals in a Eurasian beaver population in south-eastern Norway between 1998 and 2019. Beaver ID, capture year and river were included as random effects. Models were ranked based on AICc.

| Variables | df | log likelihood | AICc | deltaAICc | Weight | **R^2^_marginal_** | **R^2^_conditional_** |
| --- | --- | --- | --- | --- | --- | --- | --- |
| 1/3/4/5/8/12 | 13 | -2160.49 | 4347.43 | 0.00 | 0.04 | 0.35 | 0.57 |
| 1/3/4/5/8/11/12 | 14 | -2159.53 | 4347.58 | 0.15 | 0.04 | 0.35 | 0.56 |
| 1/3/4/8/12 | 12 | -2161.87 | 4348.12 | 0.69 | 0.03 | 0.35 | 0.57 |
| 1/3/4/8/11/12 | 13 | -2160.94 | 4348.33 | 0.90 | 0.03 | 0.35 | 0.56 |
| 1/3/4/5/8/9/12 | 14 | -2159.98 | 4348.47 | 1.04 | 0.03 | 0.35 | 0.57 |
| 1/3/4/5/7/8/12 | 14 | -2160.03 | 4348.58 | 1.15 | 0.02 | 0.35 | 0.57 |
| 1/3/4/5/7/8/11/12 | 15 | -2159.01 | 4348.61 | 1.18 | 0.02 | 0.35 | 0.56 |
| 1/3/4/5/8/9/11/12 | 15 | -2159.02 | 4348.63 | 1.20 | 0.02 | 0.35 | 0.56 |
| 1/3/4/5/8/10/12 | 14 | -2160.22 | 4348.96 | 1.53 | 0.02 | 0.35 | 0.57 |
| 1/3/4/5/8/10/11/12 | 15 | -2159.26 | 4349.10 | 1.67 | 0.02 | 0.35 | 0.56 |
| 1/3/4/8/9/12 | 13 | -2161.36 | 4349.18 | 1.75 | 0.02 | 0.35 | 0.57 |
| 1/4/5/8/12 | 12 | -2162.47 | 4349.33 | 1.90 | 0.02 | 0.34 | 0.56 |
| 1/3/4/8/9/11/12 | 14 | -2160.44 | 4349.39 | 1.96 | 0.02 | 0.35 | 0.56 |
| 1/3/4/8/10/12 | 13 | -2161.48 | 4349.42 | 1.99 | 0.02 | 0.35 | 0.57 |
| 1/3/4/7/8/12 | 13 | -2161.49 | 4349.43 | 2.01 | 0.02 | 0.34 | 0.57 |
| 1/3/4/5/7/8/9/12 | 15 | -2159.44 | 4349.47 | 2.04 | 0.02 | 0.35 | 0.57 |
| 1/2/3/4/5/8/12 | 14 | -2160.49 | 4349.50 | 2.07 | 0.02 | 0.35 | 0.57 |
| 1/3/4/5/7/8/9/11/12 | 16 | -2158.41 | 4349.50 | 2.07 | 0.02 | 0.35 | 0.56 |
| 1/3/4/7/8/11/12 | 14 | -2160.51 | 4349.54 | 2.11 | 0.02 | 0.34 | 0.56 |
| 1/3/4/8/10/11/12 | 14 | -2160.55 | 4349.61 | 2.18 | 0.01 | 0.35 | 0.56 |
| 1/2/3/4/5/8/11/12 | 15 | -2159.52 | 4349.63 | 2.20 | 0.01 | 0.35 | 0.56 |
| 1/2/3/4/5/8/12/13 | 15 | -2159.56 | 4349.70 | 2.27 | 0.01 | 0.35 | 0.57 |
| 1/2/3/4/5/8/11/12/13 | 16 | -2158.66 | 4349.99 | 2.56 | 0.01 | 0.35 | 0.57 |
| 1/3/4/5/8/9/10/12 | 15 | -2159.70 | 4349.99 | 2.56 | 0.01 | 0.35 | 0.57 |
| 1/3/5/8/11 | 12 | -2162.82 | 4350.02 | 2.59 | 0.01 | 0.34 | 0.56 |
| 1/3/4/5/7/8/10/12 | 15 | -2159.75 | 4350.09 | 2.66 | 0.01 | 0.35 | 0.57 |
| 1/3/4/5/7/8/10/11/12 | 16 | -2158.72 | 4350.11 | 2.68 | 0.01 | 0.35 | 0.56 |
| 1/3/5/8 | 11 | -2163.91 | 4350.13 | 2.71 | 0.01 | 0.34 | 0.56 |
| 1/3/4/5/8/9/10/11/12 | 16 | -2158.74 | 4350.14 | 2.72 | 0.01 | 0.35 | 0.56 |
| 1/2/3/4/8/12 | 13 | -2161.87 | 4350.18 | 2.75 | 0.01 | 0.35 | 0.57 |
| 1/2/3/4/5/8/13 | 14 | -2160.84 | 4350.20 | 2.77 | 0.01 | 0.35 | 0.57 |
| 1/4/5/8/9/12 | 13 | -2161.89 | 4350.23 | 2.80 | 0.01 | 0.34 | 0.56 |
| 1/4/8/12 | 11 | -2163.96 | 4350.25 | 2.82 | 0.01 | 0.34 | 0.56 |
| 1/2/4/5/8/12/13 | 14 | -2160.88 | 4350.28 | 2.86 | 0.01 | 0.35 | 0.57 |
| 1/2/3/4/5/7/8/11/12 | 16 | -2158.83 | 4350.33 | 2.91 | 0.01 | 0.35 | 0.56 |
| 1/2/4/5/8/12 | 13 | -2161.95 | 4350.34 | 2.91 | 0.01 | 0.34 | 0.57 |
| 1/2/3/4/8/11/12 | 14 | -2160.92 | 4350.35 | 2.92 | 0.01 | 0.35 | 0.56 |
| 1/3/4/7/8/9/12 | 14 | -2160.92 | 4350.36 | 2.93 | 0.01 | 0.34 | 0.57 |
| 1/2/3/4/5/8/11/13 | 15 | -2159.91 | 4350.40 | 2.98 | 0.01 | 0.35 | 0.57 |
| 1/2/4/5/8/13 | 13 | -2161.99 | 4350.44 | 3.01 | 0.01 | 0.34 | 0.57 |
| 1/4/5/8/11/12 | 13 | -2162.00 | 4350.46 | 3.03 | 0.01 | 0.34 | 0.56 |
| 1/3/4/7/8/9/11/12 | 15 | -2159.93 | 4350.46 | 3.03 | 0.01 | 0.35 | 0.56 |
| 1/3/4/8/9/10/12 | 14 | -2160.97 | 4350.46 | 3.03 | 0.01 | 0.35 | 0.57 |
| 1/2/3/4/5/7/8/12 | 15 | -2159.95 | 4350.48 | 3.06 | 0.01 | 0.35 | 0.57 |
| 1/2/3/4/5/8/9/12 | 15 | -2159.98 | 4350.55 | 3.12 | 0.01 | 0.35 | 0.57 |
| 1/2/3/4/5/8/9/12/13 | 16 | -2158.99 | 4350.65 | 3.22 | 0.01 | 0.35 | 0.57 |
| 1/3/4/8/9/10/11/12 | 15 | -2160.04 | 4350.67 | 3.24 | 0.01 | 0.35 | 0.56 |
| 1/2/3/4/5/8/9/11/12 | 16 | -2159.01 | 4350.69 | 3.27 | 0.01 | 0.35 | 0.56 |
| 1/3/4/7/8/10/12 | 14 | -2161.09 | 4350.70 | 3.27 | 0.01 | 0.34 | 0.57 |
| 1/4/5/8/10/12 | 13 | -2162.15 | 4350.75 | 3.32 | 0.01 | 0.34 | 0.56 |
| 1/3/4/7/8/10/11/12 | 15 | -2160.10 | 4350.79 | 3.36 | 0.01 | 0.34 | 0.56 |
| 1/3/5/8/9/11 | 13 | -2162.22 | 4350.88 | 3.45 | 0.01 | 0.35 | 0.56 |
| 1/2/3/4/5/7/8/12/13 | 16 | -2159.12 | 4350.91 | 3.48 | 0.01 | 0.35 | 0.57 |
| 1/2/3/4/5/7/8/11/12/13 | 17 | -2158.10 | 4350.96 | 3.53 | 0.01 | 0.35 | 0.56 |
| 1/2/3/4/5/8/9/11/12/13 | 17 | -2158.10 | 4350.96 | 3.54 | 0.01 | 0.35 | 0.57 |
| 1/3/4/5/7/8/9/10/12 | 16 | -2159.15 | 4350.97 | 3.54 | 0.01 | 0.35 | 0.57 |
| 1/3/5/8/9 | 12 | -2163.30 | 4350.98 | 3.55 | 0.01 | 0.34 | 0.56 |
| 1/2/3/4/5/8/9/13 | 15 | -2160.20 | 4350.99 | 3.56 | 0.01 | 0.35 | 0.57 |
| 1/3/4/5/7/8/9/10/11/12 | 17 | -2158.12 | 4350.99 | 3.56 | 0.01 | 0.35 | 0.56 |
| 1/2/4/5/8/11/12 | 14 | -2161.25 | 4351.03 | 3.60 | 0.01 | 0.35 | 0.56 |
| 1/4/5/7/8/12 | 13 | -2162.29 | 4351.03 | 3.60 | 0.01 | 0.34 | 0.56 |
| 1/2/3/4/5/8/10/12 | 15 | -2160.22 | 4351.03 | 3.60 | 0.01 | 0.35 | 0.57 |
| 1/2/3/4/8/12/13 | 14 | -2161.28 | 4351.07 | 3.64 | 0.01 | 0.35 | 0.57 |
| 1/2/4/5/8/11/12/13 | 15 | -2160.25 | 4351.09 | 3.66 | 0.01 | 0.35 | 0.57 |
| 1/2/4/8/12 | 12 | -2163.36 | 4351.10 | 3.67 | 0.01 | 0.34 | 0.57 |
| 1/2/4/5/8/11/13 | 14 | -2161.31 | 4351.14 | 3.71 | 0.01 | 0.35 | 0.56 |
| 1/4/8/9/12 | 12 | -2163.39 | 4351.17 | 3.74 | 0.01 | 0.34 | 0.56 |
| 1/2/3/4/5/8/10/11/12 | 16 | -2159.25 | 4351.17 | 3.75 | 0.01 | 0.35 | 0.56 |
| 1/2/4/5/7/8/12 | 14 | -2161.33 | 4351.18 | 3.76 | 0.01 | 0.34 | 0.56 |
| 1/3/5/7/8/11 | 13 | -2162.38 | 4351.20 | 3.77 | 0.01 | 0.34 | 0.55 |
| 1/2/4/5/8/9/12/13 | 15 | -2160.31 | 4351.20 | 3.78 | 0.01 | 0.35 | 0.57 |
| 1/2/4/5/8/9/13 | 14 | -2161.34 | 4351.20 | 3.78 | 0.01 | 0.35 | 0.57 |
| 1/2/3/4/5/8/9/11/13 | 16 | -2159.27 | 4351.22 | 3.79 | 0.01 | 0.35 | 0.57 |
| 1/2/3/4/8/9/12 | 14 | -2161.36 | 4351.24 | 3.82 | 0.01 | 0.35 | 0.57 |
| 1/2/3/4/7/8/11/12 | 15 | -2160.33 | 4351.24 | 3.82 | 0.01 | 0.34 | 0.56 |
| 1/2/3/4/5/7/8/9/11/12 | 17 | -2158.25 | 4351.26 | 3.83 | 0.01 | 0.35 | 0.56 |
| 1/2/3/4/5/8/10/12/13 | 16 | -2159.32 | 4351.31 | 3.89 | 0.01 | 0.35 | 0.57 |
| 1/2/3/4/7/8/12 | 14 | -2161.40 | 4351.32 | 3.89 | 0.01 | 0.34 | 0.57 |
| 1/4/5/8/9/11/12 | 14 | -2161.42 | 4351.35 | 3.93 | 0.01 | 0.34 | 0.56 |
| 1/2/4/5/8/9/12 | 14 | -2161.42 | 4351.36 | 3.93 | 0.01 | 0.35 | 0.57 |
| 1/2/3/4/8/11/12/13 | 15 | -2160.39 | 4351.37 | 3.95 | 0.01 | 0.35 | 0.57 |
| 1/2/4/5/7/8/12/13 | 15 | -2160.40 | 4351.39 | 3.96 | 0.01 | 0.34 | 0.57 |
| 1/2/3/4/5/7/8/9/12 | 16 | -2159.36 | 4351.40 | 3.97 | 0.01 | 0.35 | 0.57 |
| 1/4/8/10/12 | 12 | -2163.51 | 4351.41 | 3.98 | 0.01 | 0.34 | 0.56 |
| 1 = Age+Age^2^, 2 = Captures, 3 = Carried telemetry device, 4 = Social status, 5 = Family group size, 7 = Origin, 8 = Season, 9 = Sex, 10 = log(Territory size), 11 = Years of monitoring, 12 = Social status:Age, 13 = Captures:Social status | | | | | |  |  |

**S7.** The model selection result for the candidate models investigating the annual reproduction in a Eurasian beaver population in south-eastern Norway between 1998 and 2018. Beaver ID, monitoring year and river were included as random effects. Models were ranked based on AICc.

| Variables | df | log likelihood | AICc | deltaAICc | weight | **R^2^_marginal_** | **R^2^_conditional_** |
| --- | --- | --- | --- | --- | --- | --- | --- |
| 2/6/7/8/10 | 9 | -249.90 | 518.27 | 0.00 | 0.06 | 0.07 | 0.14 |
| 2/5/6/7/8/10 | 10 | -248.94 | 518.47 | 0.20 | 0.05 | 0.08 | 0.13 |
| 6/7/8/10 | 8 | -251.13 | 518.64 | 0.37 | 0.05 | 0.06 | 0.14 |
| 1/6/7/8/9/10 | 10 | -249.04 | 518.66 | 0.39 | 0.05 | 0.09 | 0.17 |
| 5/6/7/8/10 | 9 | -250.12 | 518.73 | 0.46 | 0.04 | 0.07 | 0.13 |
| 1/4/6/7/8/9/10 | 11 | -248.09 | 518.88 | 0.61 | 0.04 | 0.09 | 0.16 |
| 2/3/6/7/8/10 | 10 | -249.27 | 519.13 | 0.86 | 0.04 | 0.08 | 0.13 |
| 1/2/6/7/8/9/10 | 11 | -248.31 | 519.32 | 1.05 | 0.03 | 0.09 | 0.16 |
| 1/5/6/7/8/9/10 | 11 | -248.40 | 519.51 | 1.24 | 0.03 | 0.09 | 0.15 |
| 3/6/7/8/10 | 9 | -250.58 | 519.64 | 1.37 | 0.03 | 0.07 | 0.14 |
| 1/2/4/6/7/8/9/10 | 12 | -247.52 | 519.88 | 1.61 | 0.02 | 0.09 | 0.16 |
| 1/4/5/6/7/8/9/10 | 12 | -247.55 | 519.94 | 1.67 | 0.02 | 0.09 | 0.15 |
| 2/4/6/7/8/10 | 10 | -249.73 | 520.04 | 1.77 | 0.02 | 0.07 | 0.13 |
| 1/3/6/7/8/9/10 | 11 | -248.69 | 520.08 | 1.81 | 0.02 | 0.09 | 0.16 |
| 2/3/5/6/7/8/10 | 11 | -248.72 | 520.14 | 1.87 | 0.02 | 0.08 | 0.13 |
| 1/2/5/6/7/8/9/10 | 12 | -247.68 | 520.18 | 1.91 | 0.02 | 0.09 | 0.15 |
| 2/4/5/6/7/8/10 | 11 | -248.80 | 520.30 | 2.03 | 0.02 | 0.08 | 0.12 |
| 4/6/7/8/10 | 9 | -250.93 | 520.33 | 2.06 | 0.02 | 0.06 | 0.14 |
| 1/2/6/7/8/10 | 10 | -249.90 | 520.38 | 2.11 | 0.02 | 0.07 | 0.14 |
| 4/5/6/7/8/10 | 10 | -249.94 | 520.47 | 2.20 | 0.02 | 0.07 | 0.12 |
| 3/5/6/7/8/10 | 10 | -249.95 | 520.49 | 2.22 | 0.02 | 0.07 | 0.13 |
| 1/2/5/6/7/8/10 | 11 | -248.91 | 520.52 | 2.25 | 0.02 | 0.08 | 0.13 |
| 1/3/4/6/7/8/9/10 | 12 | -247.88 | 520.59 | 2.32 | 0.02 | 0.09 | 0.16 |
| 1/2/3/6/7/8/9/10 | 12 | -247.90 | 520.63 | 2.36 | 0.02 | 0.09 | 0.16 |
| 5/7/8/10 | 8 | -252.13 | 520.64 | 2.37 | 0.02 | 0.05 | 0.12 |
| 1/5/6/7/8/10 | 10 | -250.08 | 520.74 | 2.47 | 0.02 | 0.07 | 0.13 |
| 1/6/7/8/10 | 9 | -251.13 | 520.74 | 2.47 | 0.02 | 0.06 | 0.14 |
| 7/8/10 | 7 | -253.23 | 520.76 | 2.49 | 0.02 | 0.04 | 0.14 |
| 2/5/7/8/10 | 9 | -251.17 | 520.82 | 2.55 | 0.02 | 0.06 | 0.12 |
| 2/7/8/10 | 8 | -252.26 | 520.89 | 2.62 | 0.01 | 0.05 | 0.13 |
| 1/2/4/5/6/7/8/9/10 | 13 | -246.99 | 520.95 | 2.68 | 0.01 | 0.10 | 0.15 |
| 2/3/4/6/7/8/10 | 11 | -249.17 | 521.05 | 2.78 | 0.01 | 0.08 | 0.13 |
| 1/7/8/9/10 | 9 | -251.35 | 521.17 | 2.90 | 0.01 | 0.07 | 0.17 |
| 1/2/3/6/7/8/10 | 11 | -249.24 | 521.18 | 2.91 | 0.01 | 0.08 | 0.13 |
| 2/3/7/8/10 | 9 | -251.36 | 521.19 | 2.92 | 0.01 | 0.06 | 0.13 |
| 3/7/8/10 | 8 | -252.44 | 521.26 | 2.99 | 0.01 | 0.05 | 0.13 |
| 1/3/5/6/7/8/9/10 | 12 | -248.27 | 521.38 | 3.11 | 0.01 | 0.09 | 0.15 |
| 3/4/6/7/8/10 | 10 | -250.44 | 521.47 | 3.20 | 0.01 | 0.07 | 0.13 |
| 1/2/3/4/6/7/8/9/10 | 13 | -247.26 | 521.50 | 3.23 | 0.01 | 0.10 | 0.15 |
| 1/3/6/7/8/10 | 10 | -250.54 | 521.66 | 3.39 | 0.01 | 0.07 | 0.13 |
| 1/5/7/8/9/10 | 10 | -250.57 | 521.73 | 3.46 | 0.01 | 0.07 | 0.15 |
| 1/3/4/5/6/7/8/9/10 | 13 | -247.49 | 521.96 | 3.69 | 0.01 | 0.09 | 0.15 |
| 1/2/3/5/6/7/8/9/10 | 13 | -247.50 | 521.98 | 3.71 | 0.01 | 0.09 | 0.15 |
| 1/3/7/8/9/10 | 10 | -250.74 | 522.06 | 3.79 | 0.01 | 0.07 | 0.16 |
| 2/3/4/5/6/7/8/10 | 12 | -248.61 | 522.06 | 3.79 | 0.01 | 0.08 | 0.12 |
| 1/2/3/5/6/7/8/10 | 12 | -248.65 | 522.13 | 3.86 | 0.01 | 0.08 | 0.12 |
| 3/5/7/8/10 | 9 | -251.83 | 522.13 | 3.86 | 0.01 | 0.06 | 0.12 |
| 1/2/4/6/7/8/10 | 11 | -249.73 | 522.16 | 3.89 | 0.01 | 0.07 | 0.13 |
| 2/3/5/7/8/10 | 10 | -250.79 | 522.17 | 3.90 | 0.01 | 0.07 | 0.12 |
| 1/2/7/8/9/10 | 10 | -250.81 | 522.20 | 3.93 | 0.01 | 0.07 | 0.16 |
| 1 = log(Age), 2 = Carried telemetry device, 3 = Family group size, 4 = Origin, 5 = Reproduced previous year, 6 = log(Territory size), 7 = Captures, 8 = Years of monitoring, 9 = Captures:log(Age), 10 = Captures:Years of monitoring | | | | | |  |  |

**S8.** The model selection result for the candidate models investigating the annual litter size in a Eurasian beaver population in south-eastern Norway between 1998 and 2018. Beaver ID, monitoring year and river were included as random effects. Models were ranked based on AICc.

| Variables | df | log likelihood | AICc | deltaAICc | weight | **R^2^_marginal_** | **R^2^_conditional_** |
| --- | --- | --- | --- | --- | --- | --- | --- |
| 1/4/6/7/8/9/10 | 11 | -415.22 | 853.15 | 0.00 | 0.12 | 0.09 | 0.21 |
| 1/6/7/8/9/10 | 10 | -416.57 | 853.72 | 0.57 | 0.09 | 0.08 | 0.22 |
| 1/4/5/6/7/8/9/10 | 12 | -414.74 | 854.31 | 1.17 | 0.07 | 0.09 | 0.20 |
| 1/7/8/9/10 | 9 | -418.03 | 854.54 | 1.40 | 0.06 | 0.08 | 0.23 |
| 1/5/6/7/8/9/10 | 11 | -416.01 | 854.71 | 1.57 | 0.06 | 0.09 | 0.21 |
| 1/3/4/6/7/8/9/10 | 12 | -414.96 | 854.74 | 1.60 | 0.06 | 0.09 | 0.20 |
| 1/2/4/6/7/8/9/10 | 12 | -414.96 | 854.75 | 1.60 | 0.06 | 0.09 | 0.21 |
| 1/4/7/8/9/10 | 10 | -417.15 | 854.89 | 1.74 | 0.05 | 0.08 | 0.22 |
| 1/3/6/7/8/9/10 | 11 | -416.19 | 855.09 | 1.94 | 0.05 | 0.09 | 0.21 |
| 1/2/6/7/8/9/10 | 11 | -416.20 | 855.10 | 1.95 | 0.05 | 0.09 | 0.22 |
| 1/5/7/8/9/10 | 10 | -417.47 | 855.53 | 2.38 | 0.04 | 0.08 | 0.21 |
| 1/3/7/8/9/10 | 10 | -417.66 | 855.90 | 2.75 | 0.03 | 0.08 | 0.22 |
| 1/2/4/5/6/7/8/9/10 | 13 | -414.49 | 855.95 | 2.80 | 0.03 | 0.09 | 0.20 |
| 1/4/5/7/8/9/10 | 11 | -416.67 | 856.05 | 2.90 | 0.03 | 0.08 | 0.21 |
| 1/2/7/8/9/10 | 10 | -417.73 | 856.05 | 2.91 | 0.03 | 0.08 | 0.23 |
| 1/2/5/6/7/8/9/10 | 12 | -415.65 | 856.13 | 2.98 | 0.03 | 0.09 | 0.20 |
| 1/2/3/4/6/7/8/9/10 | 13 | -414.65 | 856.27 | 3.13 | 0.03 | 0.10 | 0.20 |
| 1/3/4/5/6/7/8/9/10 | 13 | -414.66 | 856.30 | 3.15 | 0.03 | 0.09 | 0.19 |
| 1/2/3/6/7/8/9/10 | 12 | -415.76 | 856.36 | 3.21 | 0.02 | 0.09 | 0.21 |
| 1/3/4/7/8/9/10 | 11 | -416.87 | 856.44 | 3.29 | 0.02 | 0.08 | 0.21 |
| 1/2/4/7/8/9/10 | 11 | -416.93 | 856.57 | 3.42 | 0.02 | 0.08 | 0.22 |
| 1/3/5/6/7/8/9/10 | 12 | -415.88 | 856.59 | 3.44 | 0.02 | 0.09 | 0.20 |
| 1/2/5/7/8/9/10 | 11 | -417.18 | 857.06 | 3.91 | 0.02 | 0.08 | 0.21 |
| 1 = log(Age), 2 = Carried telemetry device, 3 = Family group size, 4 = Origin, 5 = Reproduced previous year, 6 = log(Territory size), 7 = Captures, 8 = Years of monitoring, 9 = Captures:log(Age), 10 = Captures:Years of monitoring | | | | | |  |  |

**S9.** The model structure for the global model investigating the annual survival in a Eurasian beaver population in south-eastern Norway between 1998 and 2018 using a continuous time capture-recapture model (Fouchet et al. 2016):

Survival ~ Captures + Years of monitoring + Carried telemetry device + Season + Sex + Social status + log(Territory size) + Family group size + Age + Immigrant + Captures:Years of monitoring + Captures:Sex + Captures:Social status + Captures:Age + Season:Sex + Social status:Age

Model terms were removed by backwards selection until the model only consisted of informative parameters:

Survival ~ Age + Social status + Family group size + Social status:Age

**S10.** The model selection result for the candidate models investigating the probability of staying dominant in a Eurasian beaver population in south-eastern Norway between 1998 and 2018. Beaver ID, monitoring year and river were included as random effects. Models were ranked based on AICc.

| Variables | df | log likelihood | AICc | deltaAICc | weight | **R^2^_marginal_** | **R^2^_conditional_** |
| --- | --- | --- | --- | --- | --- | --- | --- |
| 1/3/6/8 | 8 | -260.70 | 537.59 | 0.00 | 0.15 | 0.20 | 0.24 |
| 1/3/4/6/8 | 9 | -260.28 | 538.80 | 1.20 | 0.08 | 0.20 | 0.25 |
| 1/3/6/7/8 | 9 | -260.56 | 539.36 | 1.77 | 0.06 | 0.20 | 0.24 |
| 1/3/5/6/8 | 9 | -260.59 | 539.42 | 1.83 | 0.06 | 0.20 | 0.24 |
| 1/2/3/6/8 | 9 | -260.60 | 539.43 | 1.84 | 0.06 | 0.20 | 0.25 |
| 1/3/6 | 7 | -262.79 | 539.73 | 2.14 | 0.05 | 0.16 | 0.24 |
| 1/3/4/6/7/8 | 10 | -259.84 | 539.97 | 2.37 | 0.04 | 0.20 | 0.24 |
| 1/2/3/6 | 8 | -261.99 | 540.16 | 2.57 | 0.04 | 0.17 | 0.25 |
| 1/3/8 | 7 | -263.08 | 540.30 | 2.71 | 0.04 | 0.17 | 0.22 |
| 1/3/4/8 | 8 | -262.15 | 540.49 | 2.90 | 0.03 | 0.18 | 0.22 |
| 1/3/4/5/6/8 | 10 | -260.13 | 540.54 | 2.95 | 0.03 | 0.20 | 0.25 |
| 1/3/6/7/8/11 | 10 | -260.17 | 540.63 | 3.03 | 0.03 | 0.20 | 0.26 |
| 1/3/4/6/7/8/9 | 11 | -259.16 | 540.66 | 3.07 | 0.03 | 0.19 | 0.23 |
| 1/2/3/4/6/8 | 10 | -260.20 | 540.70 | 3.10 | 0.03 | 0.20 | 0.25 |
| 1/3/6/7/8/9 | 10 | -260.25 | 540.79 | 3.19 | 0.03 | 0.19 | 0.23 |
| 1/2/3/6/7/8 | 10 | -260.31 | 540.92 | 3.32 | 0.03 | 0.20 | 0.25 |
| 1/3/4/6/7/8/9/11 | 12 | -258.33 | 541.06 | 3.47 | 0.03 | 0.20 | 0.25 |
| 1/3/6/7/8/9/11 | 11 | -259.41 | 541.16 | 3.57 | 0.02 | 0.20 | 0.25 |
| 1/3/5/6/7/8 | 10 | -260.48 | 541.26 | 3.66 | 0.02 | 0.20 | 0.24 |
| 1/2/3/5/6/8 | 10 | -260.50 | 541.29 | 3.70 | 0.02 | 0.20 | 0.25 |
| 1/2/3/4/6/7/8 | 11 | -259.53 | 541.41 | 3.81 | 0.02 | 0.20 | 0.25 |
| 1/3/4/6/7/8/11 | 11 | -259.53 | 541.41 | 3.81 | 0.02 | 0.21 | 0.26 |
| 1/2/3/6/7/8/9/10 | 12 | -258.54 | 541.49 | 3.90 | 0.02 | 0.22 | 0.32 |
| 1/3/5/6 | 8 | -262.68 | 541.54 | 3.95 | 0.02 | 0.16 | 0.24 |
| 1/2/3/6/7 | 9 | -261.66 | 541.56 | 3.96 | 0.02 | 0.17 | 0.25 |
| 1 = Age, 2 = Carried telemetry device, 3 = Family, 4 = Origin, 5 = Sex, 6 = log(Territory size), 7 = Captures, 8 = Years of monitoring, 9 = Captures:Age, 10 = Captures:Carried telemetry device, 11 = Captures:Years of monitoring | | | | | |  |  |
